# Construction of a prognostic model via WGCNA combined with the LASSO algorithm for stomach adenocarcinoma patients

**DOI:** 10.3389/fgene.2024.1418818

**Published:** 2024-08-07

**Authors:** Zi-duo Huang, Wen-hua Ran, Guo-zhu Wang

**Affiliations:** ^1^ Department of General Surgery, Qianjiang Central Hospital of Chongqing, Chongqing, China; ^2^ Department of General Surgery, Nanjing First Hospital, Nanjing Medical University, Nanjing, Jiangsu, China

**Keywords:** stomach adenocarcinoma, prognostic signature, weighted gene co-expression network analysis, least absolute shrinkage and selection operator, mediating analysis

## Abstract

**Objective:**

This study aimed to identify prognostic signatures to predict the prognosis of patients with stomach adenocarcinoma (STAD), which is necessary to improve poor prognosis and offer possible treatment strategies for STAD patients.

**Methods:**

The overlapping genes between the key model genes that were screened by the weighted gene co-expression network analysis (WGCNA) and differentially expressed genes (DEGs) whose expression was different with significance between normal and tumor tissues were extracted to serve as co-expression genes. Then, enrichment analysis was performed on these genes. Furthermore, the least absolute shrinkage and selection operator (LASSO) regression was performed to screen the hub genes among overlapping genes. Finally, we constructed a model to explore the influence of polygenic risk scores on the survival probability of patients with STAD, and interaction effect and mediating analyses were also performed.

**Results:**

DEGs included 2,899 upregulated genes and 2,896 downregulated genes. After crossing the DEGs and light-yellow module genes that were obtained by WGCNA, a total of 39 overlapping genes were extracted. The gene enrichment analysis revealed that these genes were enriched in the prion diseases, biosynthesis of unsaturated fatty acids, RNA metabolic process, hydrolase activity, etc. PIP5K1P1, PTTG3P, and SNORD15B were determined by LASSO-Cox. The prognostic prediction of the three-gene model was established. The Cox regression analysis showed that the comprehensive risk score for three genes was an independent prognosis factor.

**Conclusion:**

PIP5K1P1, PTTG3P, and SNORD15B are related to the prognosis and overall survival of patients. The three-gene risk model constructed has independent prognosis predictive ability for STAD.

## 1 Introduction

Stomach adenocarcinoma (STAD), as a highly molecularly and phenotypically heterogeneous gastrointestinal disease, has become the fifth cancer with high incidence globally after lung, breast, colorectal, and prostate cancers. The number of new cases is increasing annually due to aging, with the incidence of STAD in men being twice as high as in women (Santoro et al., 2014; Bray et al., 2018; Eusebi et al., 2020; Smyth et al., 2020). Although a variety of treatment methods, including surgical resection, immunotherapy, and chemotherapy, have been applied to STAD, the prognosis of patients is still unfavorable and the survival rate of patients with STAD is only 30%. The poor prognosis of STAD patients may be attributed to several reasons. First, STAD patients are hard to distinguish at an early stage due to the absence of obvious symptoms. Second, most of the patients were in the middle or advanced stages when they were diagnosed, and tumor cells have a strong infiltrate and migrate capacity (Chiurillo, 2015), which made the treatment difficult. Third is a lack of understanding of STAD initiation and development, which made us fail to provide timely guidance for STAD treatment. It follows that the symptoms of STAD and diagnosis time of patients cannot be controlled. However, we can extend our understanding of STAD development and improve the patient’s prognosis as much as possible.

Previous studies have shown that the process, development, and prognosis of STAD were related to some gene expressions, including MKI67, PLK1, COL1A1, TPX2, COL1A2, SPP1, LCP1, FN1, COL1A1, and SERPINE1 (Huang et al., 2020; Zeng Q. et al., 2021; Zhao et al., 2021). These biomarkers may be promising targets for the treatment of STAD, which are beneficial to improving the prognosis of patients with STAD. Therefore, it is urgent to find more novel biomarkers to reveal the potential pathogenic mechanisms, provide innovative therapeutic strategies, and prolong the survival time of patients with STAD. In recent years, microarray technology, united with bioinformatics analysis, has emerged as an effective tool for mining cancer-related biomarkers. Two main methods have been used for biomarker identification, namely, differentially expressed gene (DEG) analysis and weighted gene co-expression network analysis (WGCNA) (Zhang and Horvath, 2005; Langfelder and Horvath, 2008). However, we believe that the application of these two methods had limitations. First, most WGCNA studies have been conducted based on the obtained DEGs rather than on all the expression profiles of biomarkers. Therefore, several important biomarkers may be omitted. In addition, it is known that the clinical traits are significantly related to the patient’s prognosis, such as tumor grade and clinical stage, and they have profound clinical value. However, most WGCNA studies have only obtained one module that significantly correlates with the clinical traits of interest or selected the key module that shows the highest correlation coefficient with certain clinical traits. Therefore, the common WGCNA did not consider the various clinical traits as much as possible. Because of these limitations, we believe that the methodology for cancer-related biomarker identification needs to be improved.

In this work, we intended to identify the hub genes associated with the initiation and development of STAD. We first obtained the DEGs between normal and STAD groups and performed the WGCNA to explore the key module. We further conducted a series of analyses to determine the key hub genes, evaluated their clinical value, and explored the penitential regulatory mechanism. What differentiates this study from previous studies is that the WGCNA was based on the whole expression profile of all genes rather than the profiles of DEGs; in addition, the key module was identified if the module showed a significant correlation with most of the clinical traits. Our analysis considered important biomarkers and significant clinical traits as much as possible at the same time, which is the innovation and superiority of this study.

## 2 Methods

### 2.1 Data collection

The RNA-seq expression profile and clinical features of STAD patients were collected from the TCGA-STAD dataset, which involved 414 STAD patients and 35 normal samples. The RNA-seq expression profile included 17,455 genes. The clinical features of STAD patients included age, tumor grade (G), distant metastasis (M stage), lymph node metastasis (N stage), topography (T stage), clinical stage, sex, overall survival (OS) status, and OS time. T stage refers to the primary tumor size and extent of adjacent tissue involvement, categorized as T1–T4 based on tumor volume and range. N stage refers to regional lymph node involvement, with N0 indicating no involvement and N1–N3 indicating increasing degrees of involvement. M stage refers to distant metastasis, with M0 indicating no distant metastasis and M1 indicating distant metastasis. Immunohistochemistry staining in STAD was analyzed using the Human Protein Atlas (HPA) database.

### 2.2 DEG identification

The RNA-seq expression data of STAD patients were normalized using the R package “limma.” The R package “limma” was then used to screen for DEGs between STAD and normal tissues, applying a *p*-value <0.01 and an absolute fold change (FC) > 1.5 in the TCGA-STAD datasets. The DEGs were presented with a volcano plot and heat maps using the ggplot2 R package (Chi et al., 2023; Miao et al., 2023).

### 2.3 Construction of co-expression gene modules by WGCNA

The WGCNA was performed to construct co-expression modules using the whole RNA-seq expression data of STAD patients. Scale independence and average connectivity degree of the network with different power values were tested. Then, genes were classified into different modules based on topological overlap matrix (TOM)-based dissimilarities. After WGCNA, the key module can be identified. It should be stated that the key module identification in this study was based on the number of clinical traits that correlated with a certain module. The module showing a significant correlation with most clinical traits was regarded as the key module. The correlation between each module and clinical features was assessed using the “Pearson” method, with a *p*-value <0.05 considered indicative of a significant correlation (Yuan et al., 2021; Du et al., 2023). Furthermore, the genes within the key module were used for further analysis.

### 2.4 Functional enrichment analysis

The overlapping genes between DEGs and key module genes were screened using the Venn analysis. Then, the Kyoto Encyclopedia of Genes and Genomes (KEGG) analyses were performed using the “clusterProfiler” R package on the overlapping genes to explore the enriched pathways. The *p*-values were adjusted by the “Benjamin and Hochberg” (BH) method. The Gene Ontology (GO) analysis on DEGs was also performed using the “clusterProfiler” R package, and cellular component (CC), biological process (BP), and molecular function (MF) terms were annotated. These terms were selected as vital when nominal *p*-value <0.05 and false discovery rate q-value <0.05.

In addition, we also explore the single hub gene enrichment analysis via gene set enrichment analysis (GSEA). Specifically, we obtained GSEA software (version 3.0) from the GSEA website (http://software.broadinstitute.org/gsea/index.jsp) and divided the samples into high-expression groups based on the expression levels of the hub gene (≥50%) and low-expression group (<50%) (Subramanian et al., 2005). Subsequently, we downloaded c2.cp.kegg.v7.4 from the Molecular Signatures database (http://www.gsea-msigdb.org/gsea/downloads.jsp) (Liberzon et al., 2011). We utilized the symbols.gmt subset to assess related pathways and molecular mechanisms based on gene expression profiles and phenotypic grouping, with a minimum gene set of 5 and a maximum gene set of 5,000, conducting 1,000 resamplings. Statistical significance was set at a *p*-value <0.05 and a false discovery rate (FDA) <0.25.

### 2.5 Screening of hub genes and survival analysis on them

Through differential expression analysis, we have obtained the significant DEGs between tumor and control groups. Through WGCNA, we have obtained the key module genes that correlate with the clinical characteristics. Next, we identified the common genes between significant DEGs and key module genes using Venn analysis. Regarding the common genes, LASSO analysis was then conducted to filter the prognosis-related genes using the “glmnet” R package (https://glmnet.stanford.edu/articles/Coxnet.html). In addition, we set up a 10-fold cross-validation to obtain optimal λ. λ is an important parameter value in LASSO analysis. A higher λ value leads to a higher L1 penalty weight, and the coefficients of those redundant variables will be compressed to 0. The retaining genes in the final optimal LASSO model were regarded as the key hub genes.

To verify the clinical value of hub genes, we performed the PCR *in vitro* to test the expression of hub genes in human gastric cancer cells and normal cells*.* The procedure of the PCR test is presented in the following section. In addition, we performed the Kaplan–Meier (KM) analysis to explore the association between these hub genes and overall survival (OS) of patients, and the survival difference between the two groups was analyzed using the log-rank test. All the patients were divided into high- and low-expression groups based on the cut-off value of each hub gene. All possible cut-off values between the lower and upper quartiles are recomputed, and the best-performing threshold is used as the optimal cut-off. The cut-off values were obtained via the “maxstat” R package.

### 2.6 Hub gene-based risk model construction and clinical value assessment

Subsequently, based on the LASSO coefficients of each non-zero gene and their mRNA expression level, we constructed a prognostic risk model using the following formula (Simon et al., 2011): risk score = A1_coef_*X1 _expression_+ A2_coef_*X2 _expression_ … + An_coef_*Xn _expression_. According to this formula, the risk score of each patient was calculated.

We first compared the difference in risk scores among groups stratified by various clinical characteristics. Then, we conducted the KM analysis on risk score to explore the association between risk score and OS both in whole populations and various subgroups stratified by age, gender, and clinical stage. To reveal the independent role of risk score on OS, univariable and multivariable Cox regression analyses were conducted on risk score and clinical characteristics. We also performed the receiver operating characteristic (ROC) analysis via the survival R package “ROC” to assess the prediction performance of the risk score on the 1-, 3-, and 5-year survival, and its prediction performance was reflected in the area under the curve (AUC) value of the ROC curve. In addition, the decision curve analysis (DCA) was conducted to evaluate the obtained clinical net benefit. Furthermore, a nomogram analysis was conducted via the R package “rms” to evaluate the performance of the risk score for predicting the patient’s survival after combining other clinical traits. In addition, the “calibrate” function of the “rsm” package was used to plot the calibration curve, which shows the comparison scatter plot of prediction and occurrence probability and evaluates the accuracy of prediction probabilities. The C-index value was calculated to evaluate the effectiveness of the nomogram.

### 2.7 Interaction and mediation analyses associated with OS

The above analyses all focused on the importance of assessment of the risk score in OS, and we further explored the potential role of clinical traits on the association between risk score and OS. We first performed the interaction analysis to evaluate the interaction effect between clinical traits and risk scores. Specifically, interaction analyses were performed using the product of the risk score, each clinical trait, and both while incorporating multifactor Cox regression analyses to test for an interaction between them. A *p*-value for interaction <0.05 was considered statistically significant. Next, we conducted the mediation effect analysis to explore the mediating effects of clinical traits on the association between risk score and OS via Moment Structure version (AMOS) 24.0 statistical software. The clinical trait interacted with the risk score on OS. The statistical significance of the mediating effects was tested using the bootstrap method. A *p*-value less than 0.05 for total effects was the basis for assessing the mediation effects.

### 2.8 Cell culture

The human AIDS SGC7901 cell line and human gastric epithelium GES-1 cell line were bought from the Shanghai Institute of Biochemistry and Cell Biology, Chinese Academy of Sciences. A549 cell lines were cultured in Roswell Park Memorial Institute 1640 Medium (Gibco, New York, United States). The culture methods were used according to the previous literature (Xu et al., 2023).

### 2.9 Quantitative real-time PCR test

The PCR test was conducted to explore the expression of hub genes in STAD cancer cells and normal cells. Total RNA was extracted using TRIzol reagent (Invitrogen, Carlsbad, CA, United States) following the manufacturer’s instructions and reverse transcribed, and quantitative real-time PCR (qPCR) was performed as described in the reported study (Wolf et al., 2022). The expression data of the gene was normalized based on the average mean of the housekeeping genes *PIP5K1P1*, *PTTG3P*, and *SNORD15* to control the quality of qPCR data. Primer sequences of *PIP5K1P1*: forward 5′- GGC​TGG​GTC​TTA​GGG​AAA​GG-3′; reverse 5′- ACT​AAG​AGC​CTT​GCT​TTC​TGC​T-3′. Primer sequences of *PTTG3P*: forward 5′- AAT​CTG​GTT​GAG​AGC​GGC​AA -3′; reverse 5′- CAG​CCC​ATC​CTT​TGT​AGC​CA -3′. Primer sequences of *SNORD15*: forward 5′- TGA​CAC​GAT​GAC​GAG​TCA​GA -3′; reverse 5′- AGG​ACA​CTT​CTG​CCA​AAG​GA -3′.

### 2.10 Analysis of immune cell infiltration levels

To investigate the relationship between the expression of the hub gene and six types of immune cells, we analyzed immune cell infiltration levels using the tumor immune estimation resource (TIMER) methods based on the TIMER2.0 database (http://timer.cistrome.org/), which is commonly used for studying immune cell infiltrates in various tumors (Li et al., 2020). The immune cells of interest included B cells, CD8^+^ T cells, CD4^+^ T cells, macrophages, neutrophils, and dendritic cells, with the relationship being adjusted for tumor purity. In addition, IOBR is a computational tool used for immuno-oncology biology research (Zeng D. et al., 2021). In this study, we employed expression profiles and utilized the R package IOBR along with the selected CIBERSORT method to calculate the scores for 22 immune-infiltrating cells per sample (Zeng D. et al., 2021). Additionally, patients with STAD were divided into two groups based on the median risk score, and levels of 22 immune cell infiltrations were compared between these two groups.

### 2.11 Statistical analysis

**FIGURE 1:**
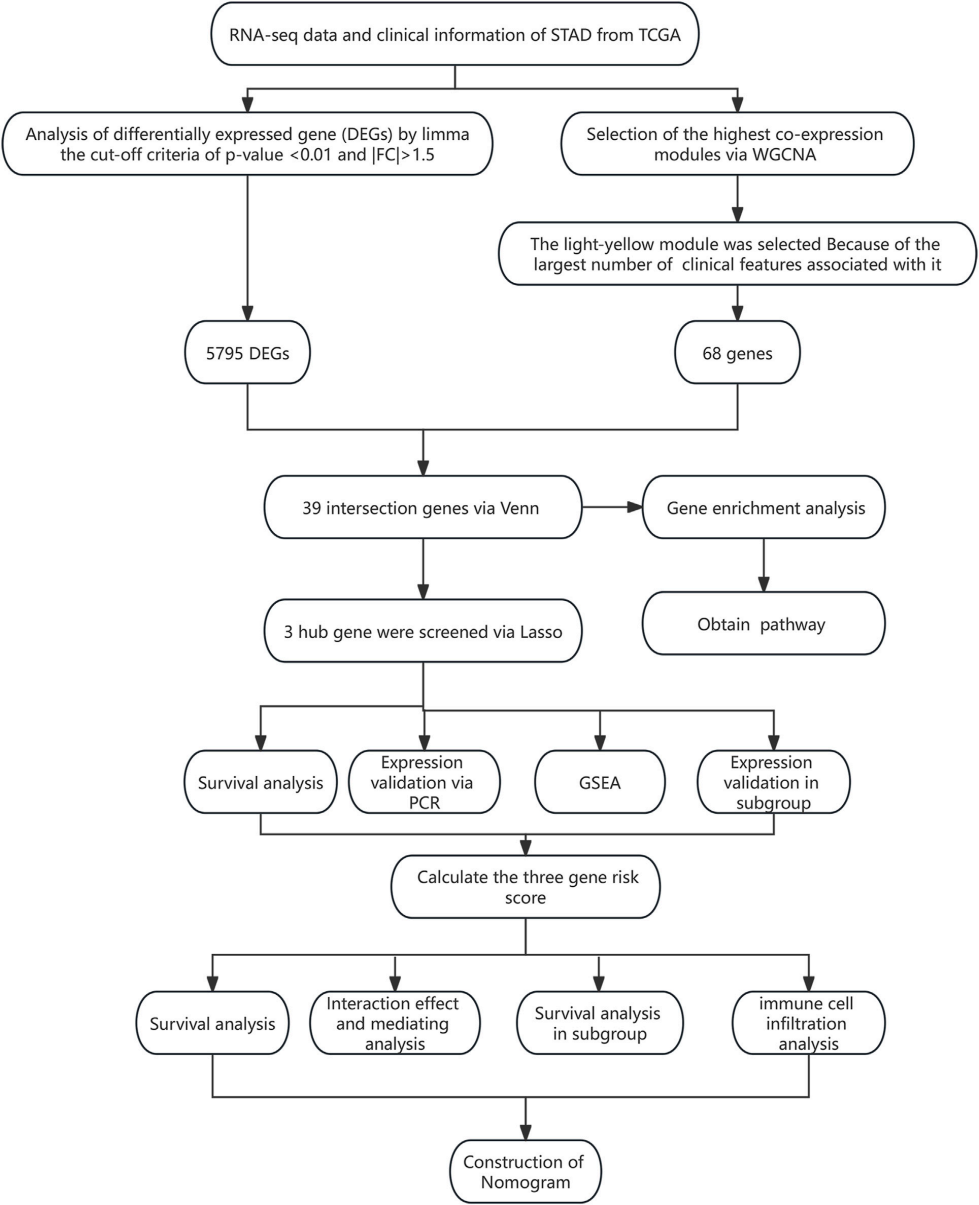
Flowchart of this study.

All statistical analyses were performed using R (version 4.0.3), AMOS (version 24), and SPSS (version 23) software. The expression differences of genes in different subgroups were analyzed using a *t*-test (2 groups) or one-way analysis of variance (ANOVA) (>2 groups). The correlation between the two variables was analyzed using the Pearson method. The survival difference between the two groups was analyzed using the log-rank test. ROC and DCA analyses were performed to evaluate the efficiency of the risk model for predicting the survival of STAD patients. The hazard ratio (HR) and 95% confidence interval (CI) were used to evaluate death risk. A *p*-value <0.05 was considered statistically significant. The research flowchart for this study is shown in Figure 1.

## 3 Results

### 3.1 Baseline characteristics

The baseline characteristics of all STAD patients are presented in Table 1. There were 267 male and 147 female patients. In addition, 226 patients aged ≥65 accounted for 54.59%, while 183 patients aged <65 accounted for 44.2%. The number of patients in G1, G2, G3, and GX was 12 (2.90%), 147 (35.51%), 246 (59.42%), and 9 (2.17%), respectively. The number of patients at M0, M1, and MX was 367 (88.65%), 27 (6.52%), and 20 (4.83%), respectively. There were 123 (29.71%) patients in N0, 111 (26.81%) patients were in N1, 80 (19.32%) patients were in N2, 82 (19.81%) were in N3, and 17 (4.11%) were in NX. The number of patients at T1, T2, T3, T4, and TX stages was 22 (5.31%), 88 (21.26%), 179 (43.24%), 116 (28.02%), and 9 (2.17%), respectively. Most of the patients were in stage III (170 cases; 41.06%) and stage II (122 cases; 29.47%), followed by stage I (57 cases; 13.77%), and the smallest number of patients was in stage IV (42 cases; 10.14%). Among all patients, 250 (60.39%) cases were alive and 121 cases (29.23%) died of STAD tumors. The patients missing overall survival time and status were excluded, and 357 STAD patients were selected for further analysis.

**TABLE 1:** Clinical characteristics of 414 STAD patients from TCGA databases.

Characteristics	N (%)
Age	
≥65	226 (54.59%)
<65	183 (44.20%)
Miss	5 (1.21%)
Grade	
G1	12 (2.90%)
G2	147 (35.51%)
G3	246 (59.42%)
GX	9 (2.17%)
M	
M0	367 (88.65%)
M1	27 (6.52%)
MX	20 (4.83%)
N	
N0	123 (29.71%)
N1	111 (26.81%)
N2	80 (19.32%)
N3	82 (19.81%)
NX	17 (4.11%)
Miss	1 (0.24%)
T	
T1	22 (5.31%)
T2	88 (21.26%)
T3	179 (43.24%)
T4	116 (28.02%)
TX	9 (2.17%)
Stage	
I	57 (13.77%)
II	122 (29.47%)
III	170 (41.06%)
IV	42 (10.14%)
Miss	23 (5.56%)
Gender	
Male	267 (64.49%)
Female	147 (35.51%)
OS	
Alive	250 (60.39%)
Dead	121 (29.23%)
Miss	43 (10.39%)

### 3.2 Identification of DEGs

There were 5,795 DEGs screened between normal and tumor groups (adj. *p*-value <0.05 and |fold change| > 1.5), including 2,899 upregulated and 2,896 downregulated genes. The DEGs were presented with a volcano map, and the top 50 upregulated and downregulated DEGs were plotted with heat maps (Supplementary Figure S1).

### 3.3 Construction of the co-expression gene model and screening of key modules

**FIGURE 2:**
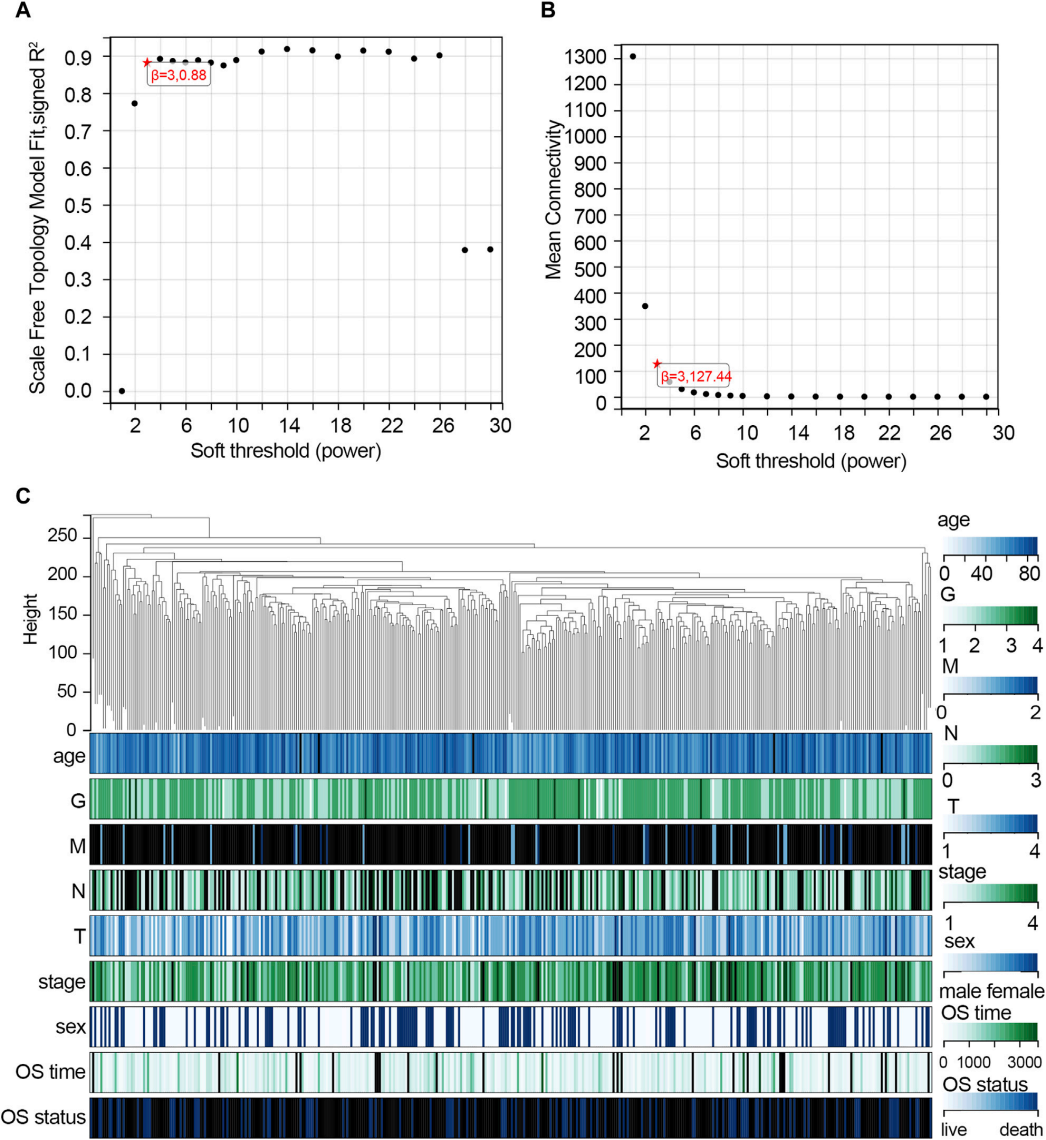
Construction of the co-expression network via WGCNA based on the TCGA-STAD dataset. **(A)** Analysis of the scale-free fit index for various soft-thresholding powers (β = 3). **(B)** Mean connectivity for various soft-thresholding powers. **(C)** Cluster map of samples with clinical features. Each vertical line represents a sample. The color depth changes of legend in the right represent the categories of each feature. Abbreviation: G, tumor grade; M, distant metastasis, N, lymph node metastasis; T, topography; OS, overall survival.

**FIGURE 3:**
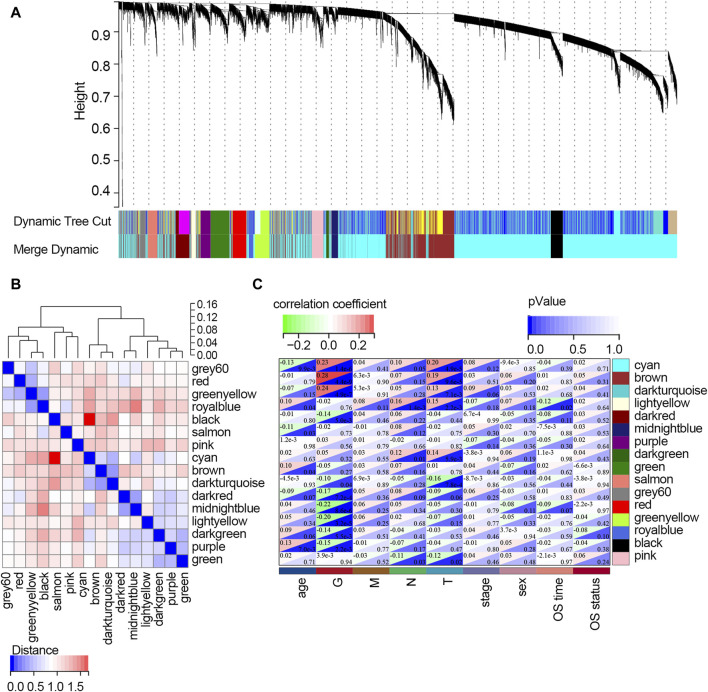
Screening of the key modules correlated with clinical traits of STAD patients via WGCNA. **(A)** Dendrogram of genes clustered based on the dissimilarity measure. Each color represents a module in the constructed gene co-expression network by WGCNA. **(B)** Heat map showing the distance between the modules. Red color represents lower overlap, and blue represents higher overlap. **(C)** Heat map showing the relationship between each module and each clinical feature. Abbreviation: G, tumor grade; M, distant metastasis, N, lymph node metastasis; T, topography; OS, overall survival.

The WGCNA was used to study the relationship between gene modules and clinical features. The results indicated that the constructed gene co-expression network approximated a scale-free topology distribution with fitting *R* ^2^ = 0.88 when soft-thresholding value β and cut height were 3 and 0.25, respectively (Figures 2A, B). A cluster map of samples with clinical features is shown in Figure 2C. Then, a gene cluster tree was presented in Figure 3A, and 16 modules were identified. Heat maps showing the distance between module genes are shown in Figure 3B.

The relationships between each module and clinical features are presented in Figure 3C. It follows that the cyan module significantly correlated with three clinical traits (age, G, and T; all *p*-values <0.05). Three modules, including brown, dark turquoise, and salmon modules, all correlated with two clinical traits (G and T; all *p*-values <0.05). The dark red model only correlated with the G trait (*p*-value <0.05). The midnight blue module only correlated with age (*p*-value <0.05). The dark green model significantly correlated with two clinical traits (N stage and T stage; all *p*-values <0.05). The light-yellow module significantly correlated with four clinical traits (age, T stage, N stage, and OS time; all *p*-values <0.05). However, the purple module and green model showed no relationship with any clinical traits. Among all the modules, the light-yellow module was finally regarded as the key module since it showed a significant correlation with most of the clinical traits. There were 68 genes within the light-yellow module.

### 3.4 Functional enrichment analyses for overlapping genes

**FIGURE 4:**
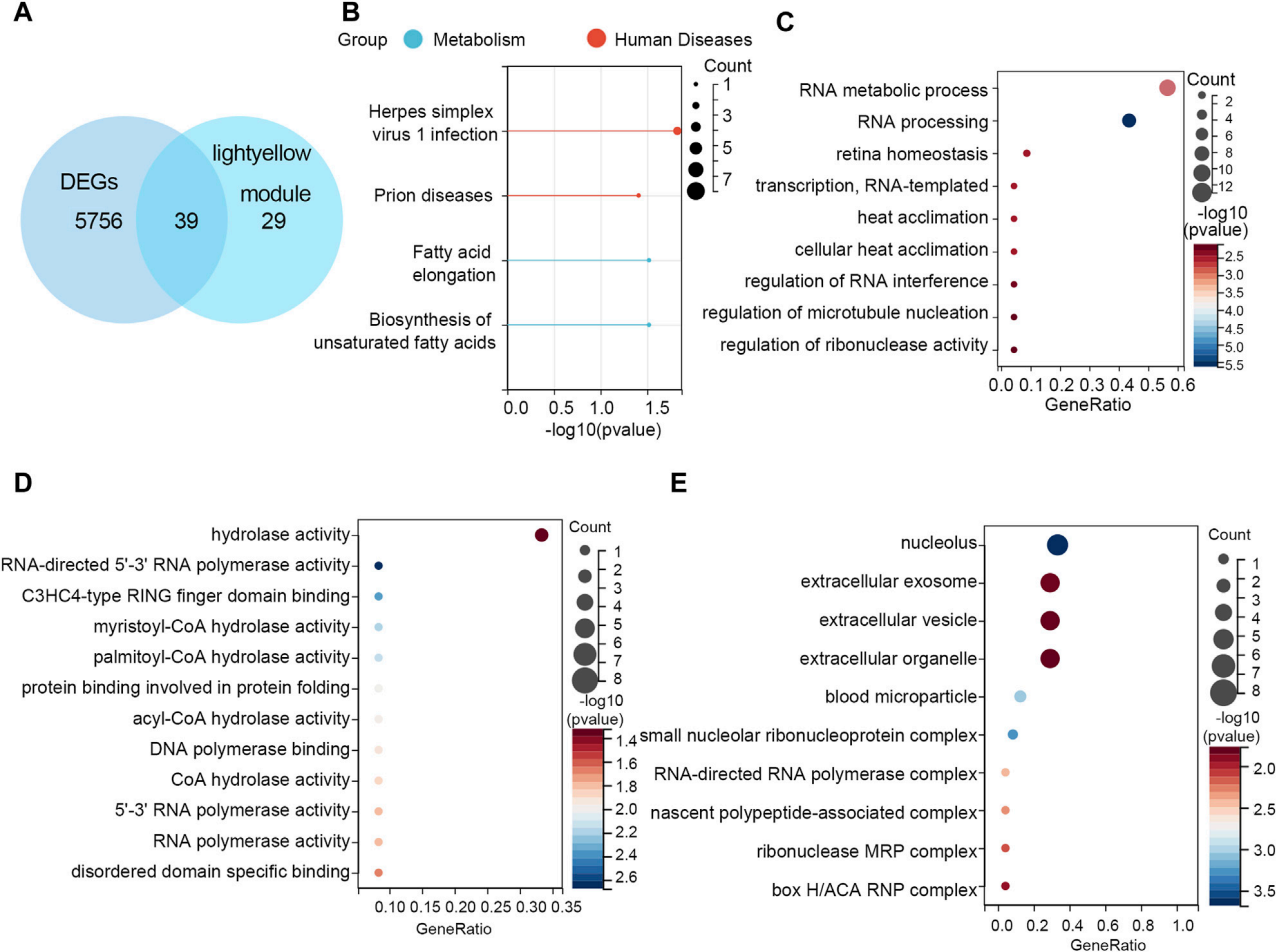
Functional enrichment analyses. **(A)** Thirty-nine overlapping genes between DEGs and the light-yellow module genes are shown in the Venn diagram. **(B)** KEGG pathway analysis on 39 genes. GO enrichment analysis on 39 genes including the **(C)** biological process, **(D)** molecular function, and **(E)** cellular component.

After crossing the DEGs and light-yellow module genes, 39 overlapping genes were extracted (Figure 4A). To further investigate the cellular functions and molecular mechanisms of overlapping genes in STAD, these genes were used to perform KEGG and GO analyses. The results of KEGG enrichment analysis revealed that 39 genes were related to the herpes simplex virus 1 infection, prion diseases, fatty acid elongation, and biosynthesis of unsaturated fatty acids (Figure 4B). Moreover, in GO enrichment analysis, the biological process contains RNA metabolic process, RNA processing retina homeostasis, transcription RNA-templated, heat acclimation, cellular heat acclimation, and regulation of RNA interference, microtubule nucleation, and ribonuclease activity (Figure 4C). The molecular function analysis indicated that 39 genes participated in hydrolase activity, RNA-directed 5′-3′ RNA polymerase activity, C3HC4-type RING finger domain binding myristoyl-CoA hydrolase activity, palmitoyl-CoA hydrolase activity, protein binding involved in protein folding, acyl-CoA hydrolase activity, DNA polymerase binding, CoA hydrolase activity, 5′-3′ RNA polymerase activity, RNA polymerase activity, and disordered domain-specific binding (Figure 4D). In cell components, these genes were engaged in the nucleolus, extracellular exosome, extracellular vesicle, extracellular organelle, blood microparticle, small nucleolar ribonucleoprotein, complex RNA-directed RNA polymerase complex, nascent polypeptide-associated complex, ribonuclease MRP complex, and box H/ACA RNP complex (Figure 4E).

### 3.5 Screening of hub gene

**FIGURE 5:**
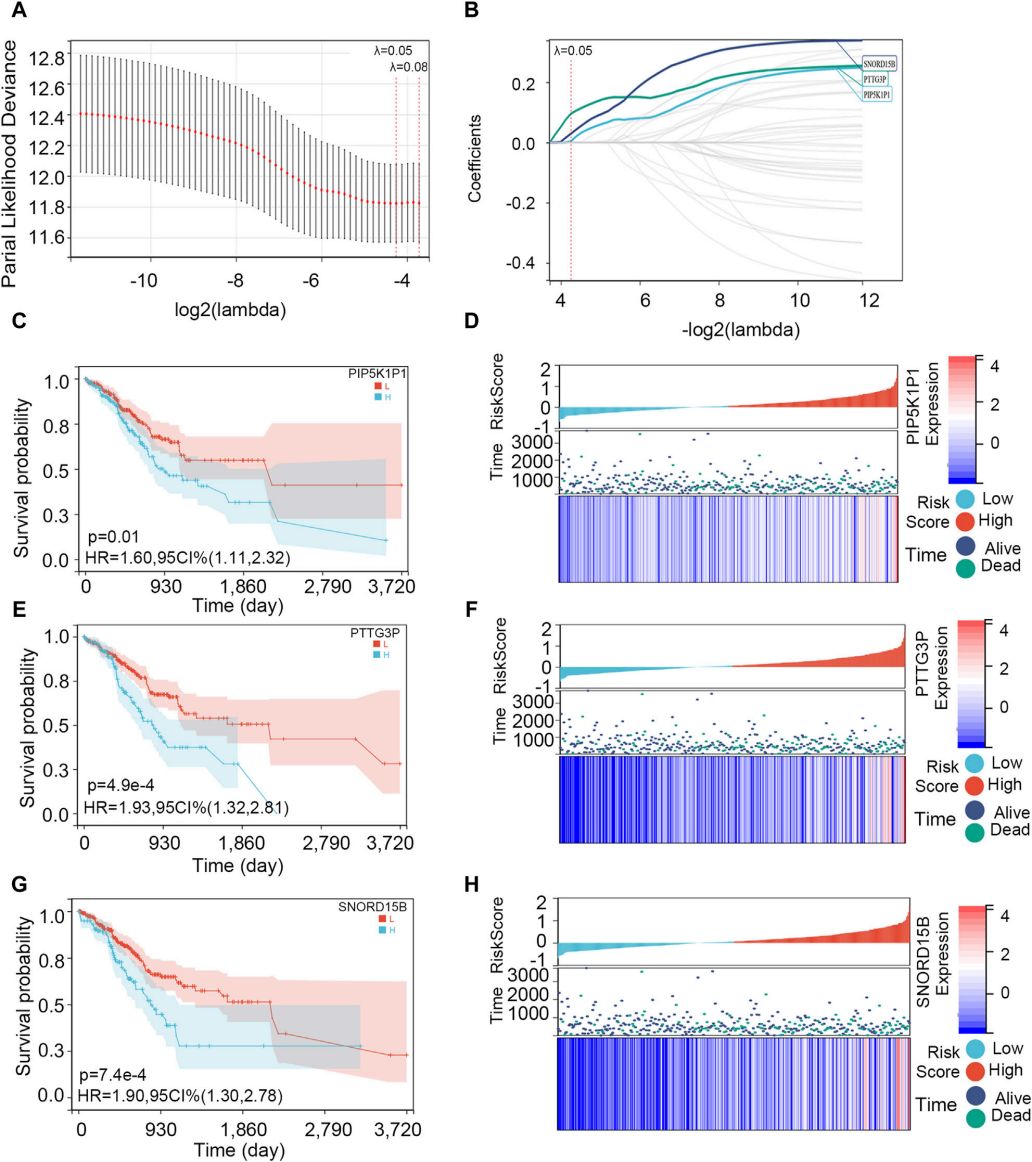
Screening of the hub genes and survival analysis on them. **(A)** Cross-validation plot for the term of penalty. **(B)** Hub genes included in the optimal LASSO model. **(C, E, G)** Association between *PIP5K1P1*, *PTTG3P*, and *SNORD15B* expressions and OS. **(D, F, H)** Visualization of *PIP5K1P1*, *PTTG3P*, and *SNORD15B* expression levels and survival condition.

The 39 overlapping genes were further enrolled in LASSO analysis (Figure 5A) to filter the redundant genes by introducing a tuning parameter (λ). When λ was minimized to 0.05, the optimal LASSO model was obtained. Finally, three hub genes, namely, *PIP5K1P1*, *PTTG3P*, and *SNORD15B*, were included in the optimal LASSO model (Figure 5B).

Then, we explored the association between three hub genes and OS in STAD patients. All the patients were divided into two groups according to their optimal cut-off values, and the optimal cut-off values of *PIP5K1P1*, *PTTG3P*, and *SNORD15B* were 0.362, −0.813, and 1.510, respectively. Next, the survival difference between the two groups was compared. Survival analysis showed that three gene expressions impacted the OS of patients with STAD (*p*-value <0.05), and the survival rate of patients in the low-expression group was higher (Figures 5C, E, G). As shown in Figures 5D, F, H, the expressions of *PIP5K1P1*, *PTTG3P*, and *SNORD15B* in dead STAD samples were higher.

### 3.6 Exploration and validation of hub gene expression

To explore the expression of hub genes in different tumors, the expression analysis in pan-cancer was performed. The results showed that *PIP5K1P1* was highly upregulated in 12 types of cancers (GBM, glioblastoma multiforme; LUAD, lung adenocarcinoma; COAD, colon adenocarcinoma; COADREAD, colorectal cancer; BRCA, breast invasive carcinoma; ESCA, esophageal carcinoma; STES, stomach and esophageal carcinoma; STAD, stomach adenocarcinoma; HNSC, head and neck squamous cell carcinoma; LUSC, lung squamous cell carcinoma; LIHC, liver hepatocellular carcinoma; and CHOL, cholangiocarcinoma) and downregulated in five types of cancers (KIRP, kidney renal papillary cell carcinoma; KIPAN, pan-kidney cohort; KIRC, kidney renal clear cell carcinoma; THCA, thyroid carcinoma; and KICH, kidney chromophobe). *PTTG3P* and *SNORD15B* were significantly upregulated in STAD and NHSC and downregulated in THCA with a statistically significant difference (Supplementary Figure S2).

We compared gene expression levels in subgroups with different clinical traits to explore the expression of three hub genes in different clinical traits. The expression of the three hub genes in different stages, N, M, G, and T, was not different. Notably, the expression of *PTTG3P* was different in gender subgroups and was higher in the female group than in the male group. The expression of *SNORD15B* differed in age subgroups and was higher in the <65 group than in the ≥65 group (Supplementary Figure S3). In addition, we also explored the expression of the hub gene at the protein level via the Human Protein Atlas Database. Supplementary Figure S4 shows that SNORD15B protein expression differs between stomach cancer tissue and normal tissue. The HPA database lacks *PTTG3P* gene expression at the protein level, and *PIP5K1P1* does not encode a protein because it is a pseudogene of *PIP5K1A*.

**FIGURE 6:**
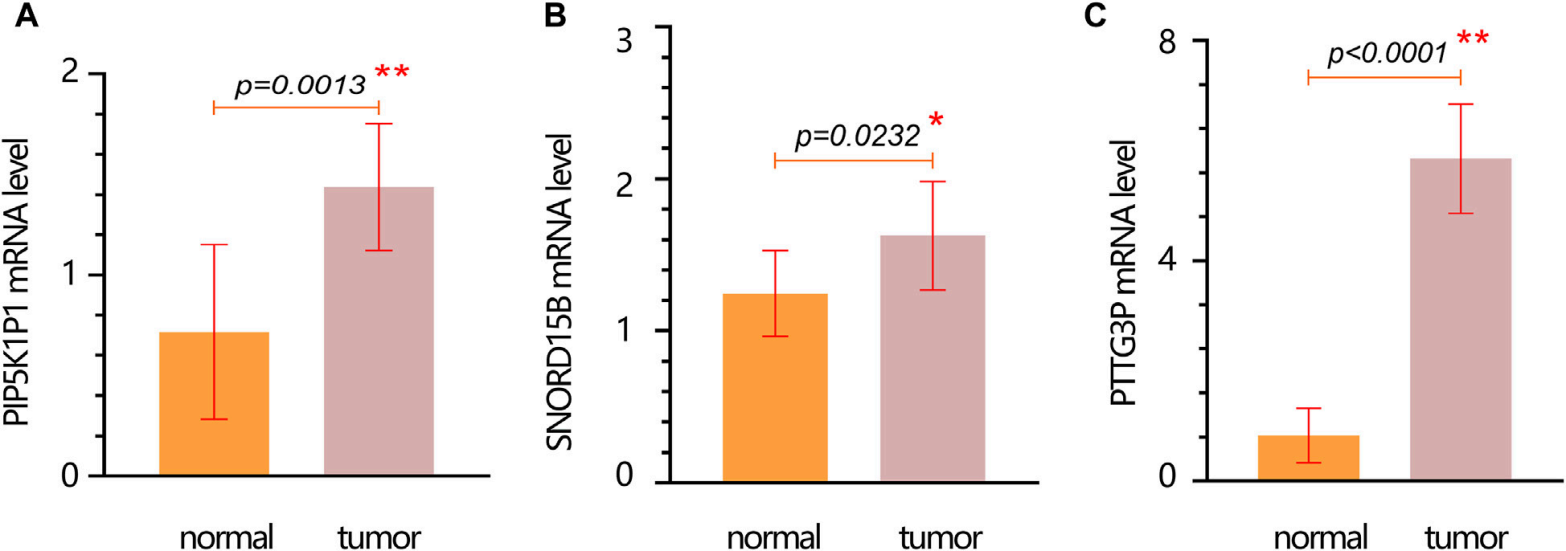
mRNA expression verification of hub genes in normal and tumor cells. **(A)** *PIP5K1P1*. **(B)** *SNORD15B*. **(C)** *PTTG3P*. Data are represented as the mean ± SD. * *p*-value < 0.05; ** *p*-value < 0.01.

We further performed the PCR test to verify the expression of hub genes. The results of PCR showed that the mRNA expression levels of *PIP5K1P1*, *PTTG3P*, and *SNORD15B* in the cancer cells were significantly higher than those in normal cells (Figures 6A–C).

### 3.7 Construction and evaluation of a three-gene signature

**FIGURE 7:**
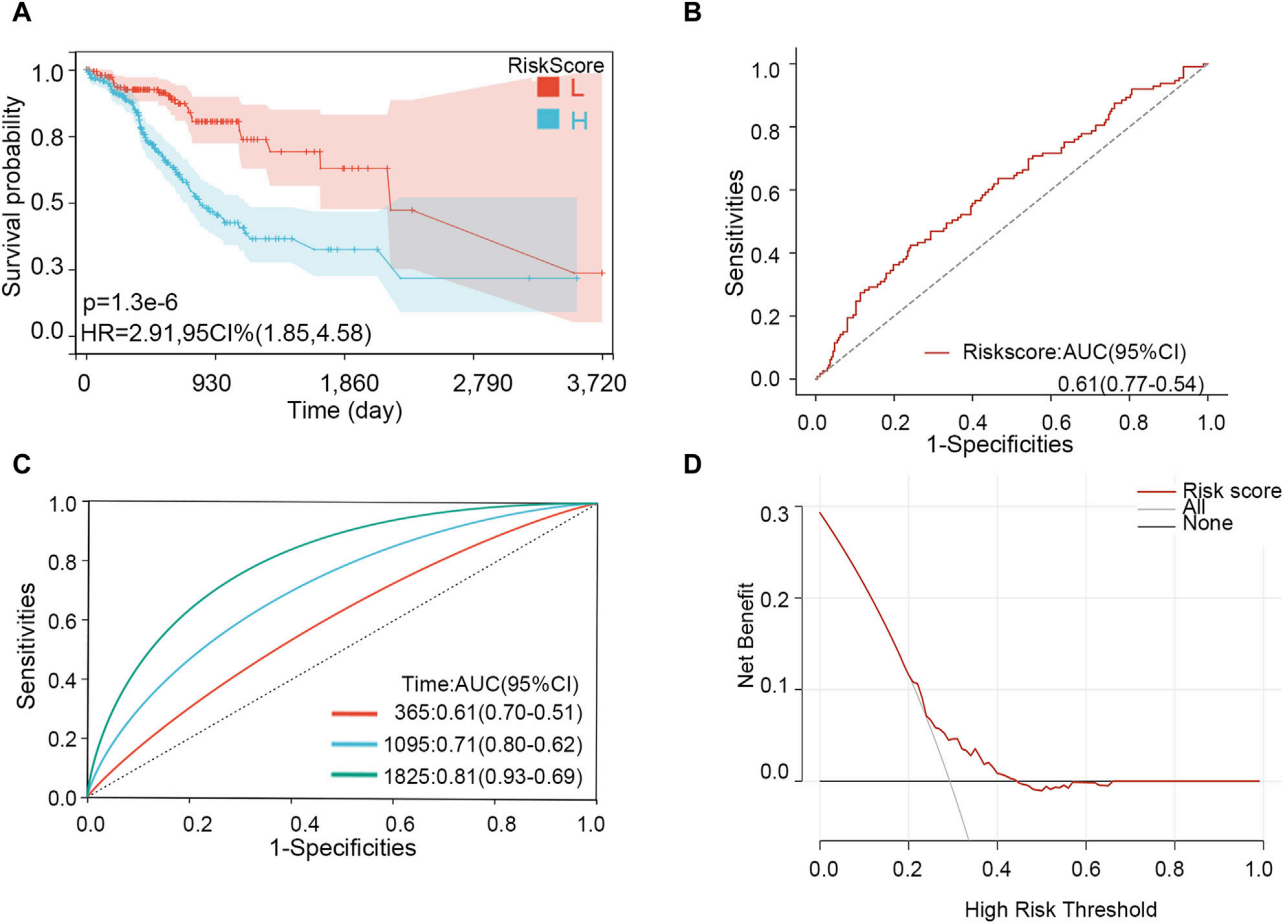
Effectiveness analysis of the risk model in STAD. **(A)** Survival analysis. **(B)** ROC analysis for predicting the survival status. **(C)** Time-dependent ROC analysis for predicting 1-, 3-, and 5-year survival. **(D)** DCA curve.

Through LASSO analysis, three biomarkers have been obtained. According to their coefficients in the LASSO model and mRNA expression level, a risk model was constructed, and the risk score of each patient was calculated according to the formula: Risk score = 0.0043*the expression level of *PIP5K1P1* + 0.096*the expression level of *PTTG3P* + 0.029*the expression level of *SNORD15B*. Survival analysis showed that the patients with a high-risk score had a low survival rate (*p*-value <0.001) (Figure 7A). The AUC value of the risk score for predicting the survival status was 0.61 (Figure 7B). The AUC values for predicting the 1-, 3-, and 5-year survival were 0.61, 0.71, and 0.81, respectively (Figure 7C). The result of DCA showed that the risk model had a good clinical net benefit in the prediction of STAD prognosis (Figure 7D). Those results indicated the model had a good predictive capability. To verify its effect on survival in different races, we performed subgroup analysis in different races. The results showed that in White and Asian populations, the high-risk group remained unfavorable for patient prognosis, which was consistent with the results in the entire population (Supplementary Figure S5) (due to the small number of Black or African American/Native Hawaiian or other Pacific Islander patients, KM analysis was not performed).

### 3.8 Exploring the prognosis value of hub genes in pan-cancer

The above results showed that three genes were related to the prognosis of patients with STAD. Therefore, we further explore the prognosis value of the three genes via survival analysis in different cancers. The result showed that *PIP5K1P1* had important significance in evaluating the prognosis of patients with some types of cancer. Those types of cancer included BLCA, KIRC, UCEC, GBM, HNSC, PCPG, READ, and STES (all *p* < 0.05, Supplementary Table S1). In addition, *PTTG3P* had significant prognosis value in patients with different cancers, including KIPAN, BRCA, KICH, KIRC, COADREAD, ESCA, LIHC, LUAD, LUSC, KIRP, and PRAD (all *p* < 0.05, Supplementary Table S2). Similarly, *SNORD15B* also had prognosis value in some types of cancer, including KICH, KIRC, LIHC, and STES (all *p* < 0.05, Supplementary Table S3). Those results indicated that three hub genes had different prognoses in different types of cancer.

### 3.9 GSEA

The GSEA was performed to explore the important pathways related to the hub gene. According to *p* < 0.05 and FDR <0.25, we found no pathway associated with the expression of *PIP5K1A* and *PTTG3P*. Four pathways associated with high expression of *SNORD15B*, including oxidative phosphorylation (ES = 0.7288 and FDR = 0.0112), RNA polymerase (ES = 0.6060 and FDR = 0.2002), folate biosynthesis (ES = 0.6978 and FDR = 0.2038), and glutathione metabolism (ES = 0.4939 and FDR = 0.2319) (Supplementary Figure S6).

### 3.10 Survival analysis of the three-gene risk score

**FIGURE 8:**
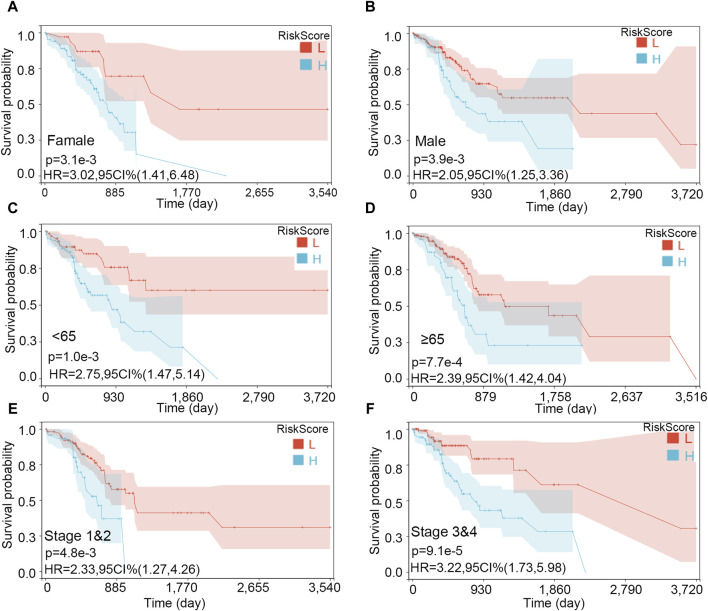
Survival analysis on the risk score in various subgroups. **(A)** Female, **(B)** male, **(C)** age <65, **(D)** age ≥65, **(E)** stages 1 and 2, and **(F)** stages 3 and 4.

The importance of the risk score in OS has already been proved. Furthermore, we explored the independent role of the risk score in OS. In the univariable and multivariate Cox analyses, the risk score was related to prognosis (*p*-value < 0.001), while other clinical traits were insignificantly associated with prognosis (Table 2). The result further indicated the importance of the three-gene model in OS. Subsequently, the KM subgroup analysis on the risk score was also performed, grouped by gender, age, and stage. The KM analysis indicated that a low-risk score was related to a longer survival time, regardless of clinical traits (Figures 8A–F, *p*-value < 0.001).

**TABLE 2:** Cox regression analysis based on the risk score and overall survival in STAD patients.

	Subject	B	*p*-value	HR	Lower limit	Upper limit	Hazard ratio (95% CI)
Univariable	Risk score	3.52	<0.001	33.87	6.22	184.54	33.87 (6.22–184.54)
	Age	0.01	0.30	1.01	0.99	1.03	1.01 (0.99–1.03)
	G	0.00	0.99	0.99	0.71	1.40	0.99 (0.71–1.40)
	M	0.02	0.94	1.02	0.64	1.63	1.02 (0.64–1.63)
	N	−0.05	0.55	0.95	0.80	1.13	0.95 (0.80–1.13)
	T	0.00	1.00	1.00	0.80	1.25	1.00 (0.80–1.25)
	STAGE	−0.04	0.73	0.96	0.77	1.20	0.96 (0.77–1.20)
	Sex	0.31	0.12	1.36	0.92	2.00	1.36 (0.92–2.00)
	Age group	0.10	0.60	1.12	0.75	1.63	1.12 (0.75–1.63)
Multivariable							
	Risk score	3.63	<0.001	37.70	6.58	215.97	37.70 (6.58–215.97)
	Age	0.02	0.34	1.02	0.98	1.05	1.02 (0.98–1.05)
	G	−0.10	0.58	0.91	0.64	1.28	0.91 (0.64–1.28)
	M	−0.02	0.95	0.98	0.60	1.62	0.98 (0.60–1.62)
	N	−0.02	0.84	0.98	0.78	1.22	0.98 (0.78–1.22)
	T	0.08	0.60	1.09	0.80	1.49	1.09 (0.80–1.49)
	STAGE	−0.04	0.83	0.96	0.67	1.38	0.96 (0.67–1.38)
	Sex	0.29	0.15	1.34	0.90	1.99	1.34 (0.90–1.99)
	Age group	−0.19	0.61	0.83	0.40	1.71	0.83 (0.40–1.71)

### 3.11 Interaction effect analysis and mediating analysis

Although no independent prognostic value of other clinical variables was found in our multivariable Cox regression, we noted that there were differences in the risk score between groups under different clinical characteristics (data not shown). Therefore, we further explored whether there was an interaction effect between clinical features and risk scores via interaction analysis. The results showed that only the N stage and clinical stage had an interaction effect with the risk score (Table 3). Because of the important role of gene marker expression in tumor progression, we further explored whether markers can affect the prognosis of patients by promoting N and clinical stages via mediation analysis. Mediation analysis showed that only the N stage played a mediating role in the correlation between the risk value and prognosis (Table 4).

**TABLE 3:** Interaction effect analysis between the different clinical characteristics and risk score in affecting the survival status.

Subject	F	*p*-value
Stage	2.94	0.02
Sex	0.47	0.51
Age	2.21	0.14
G	1.71	0.16
M	1.22	0.31
N	4.44	0.001
T	1.95	0.19

**TABLE 4:** Mediating analysis for the relationships between underlying variables.

Path	Coefficient	se	*p*-value	CI [2.5%]	CI [97.5%]	Sig
Stage as the mediator						
Stage ∼ risk score	−0.09	0.36	0.81	−0.80	0.62	No
OS time ∼ stage	−28.84	20.19	0.15	−68.48	10.80	No
Total	−735.97	201.74	0.00	−1,132.00	−339.94	Yes
Direct	−738.57	201.60	0.00	−1,134.31	−342.82	Yes
Indirect	2.60	15.58	0.80	−17.47	47.98	No
N as the mediator						
N ∼ risk score	−0.94	0.47	0.05	−1.87	−0.01	Yes
OS time ∼ N	−46.52	15.42	0.00	−76.78	−16.26	Yes
Total	−735.97	201.74	0.00	−1,132.00	−339.94	Yes
Direct	−783.65	200.96	0.00	−1,178.14	−389.16	Yes
Indirect	47.68	27.69	0.04	4.70	116.59	Yes

**FIGURE 9:**
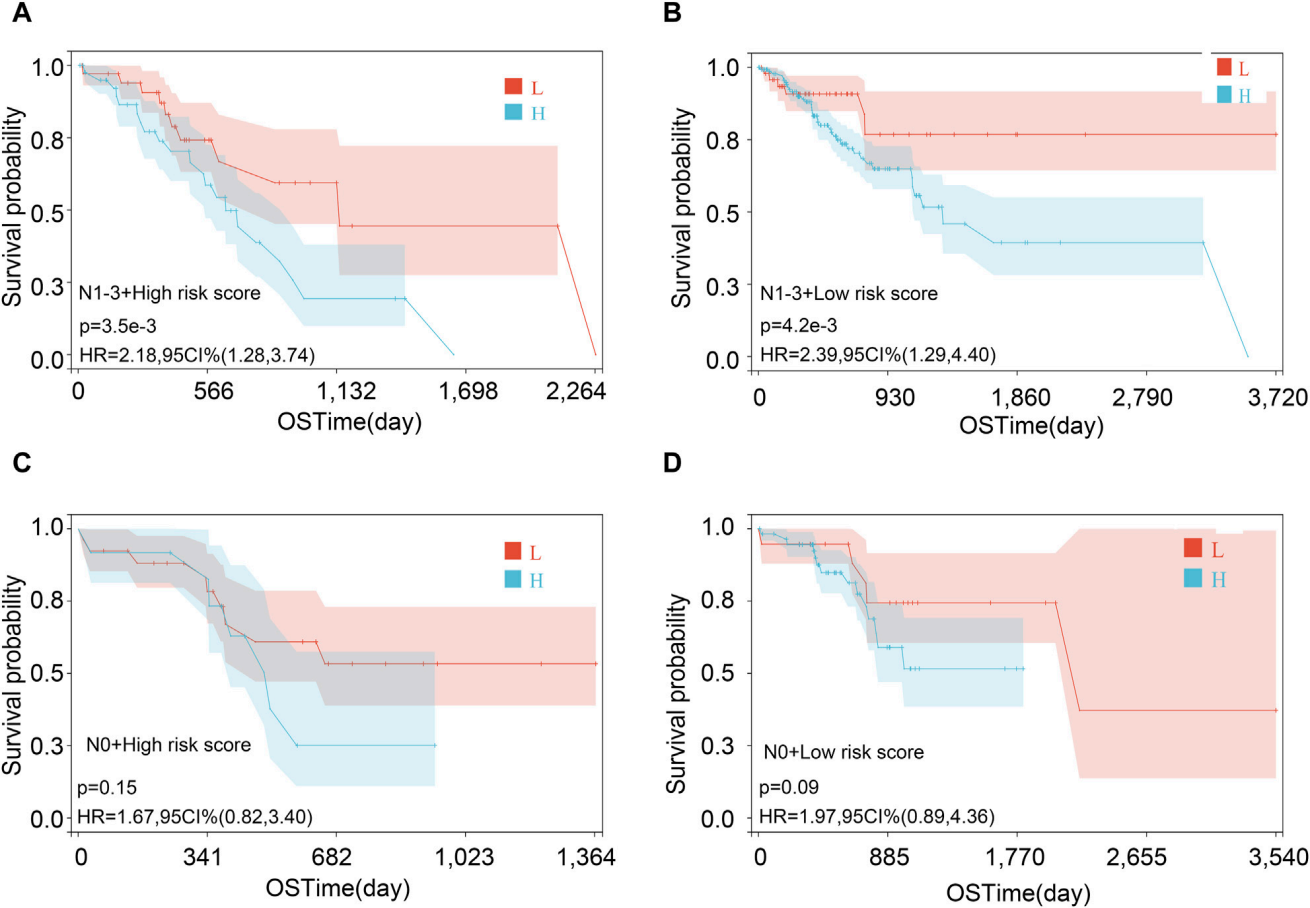
Survival analysis in various subgroups after combining risk score and lymph node metastasis status. **(A)** N1-3 and high-risk score; **(B)** N1-3 and low-risk score; **(C)** N0 and high-risk score; and **(D)** N0 and low-risk score.

Then, we performed the KM analysis in four groups, namely, N1–3 and high-risk score, N1–3 and low-risk score, N0 and high-risk score, and N0 and low-risk score for exploring the precise crowd. We found that the risk score mainly correlated with the prognosis in patients with lymph node metastasis (Figures 9A–D).

### 3.12 Analysis of immune cell infiltration levels

The correlations between immune cell infiltration levels and expression of the hub genes in STAD were explored via TIMER. The results showed that the expression of hub genes had a different association with the infiltration level of the different types of immune cells. Notably, the expression of *PTTG3P* had a positive association with the infiltration level of the CD8+ T cell (r = 0.1 and *p* < 0.001), macrophage (r = 0.146 and *p* < 0.001), and neutrophil (r = 0.18 and *p* < 0.001). The abundance of 22 immune cell infiltrates calculated by the CIBERSORT method showed that the levels of eosinophils and neutrophils were significantly higher in the high-risk group than in the low-risk group (Supplementary Figure S7).

### 3.13 Construction of a nomogram model

**FIGURE 10:**
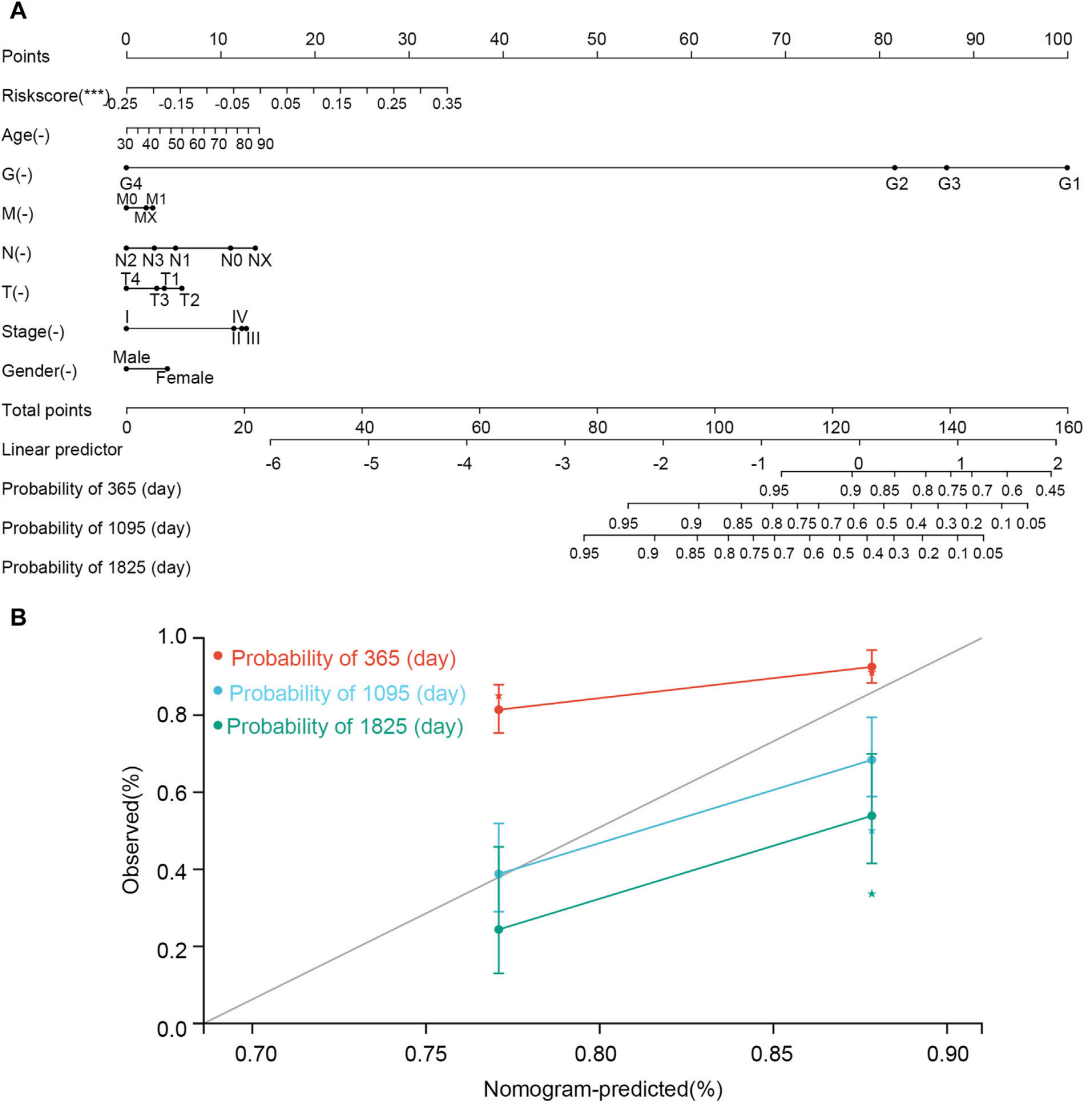
Nomogram established using clinical features and risk scores. **(A)** Nomogram. **(B)** correction curve. In the nomogram, the line corresponding to each variable is marked with a scale, which represents the range of the variable, and the length of the line segment reflects the contribution of the factor to the clinical outcome event. The point represents the individual score corresponding to each variable under different values. Total point represents the total score of the sum of the individual scores corresponding to all variables. Prediction probability represents the risk of suffering from STAD.

The nomogram was constructed according to various clinical traits and risk scores to assess the performance for predicting the 1-, 3-, and 5-year survival probability (Figure 10). The C-index was 0.660, hazard ratio was 95% CI (0.598–0.721), and *p*-value was 3.785e-07. The nomogram showed that tumor grade and risk scores had higher contributions to survival than other features. The result also indicated that the model had an excellent ability to assess disease progression and predict survival for every STAD patient.

## 4 Discussion

STAD is a common cancer with a high mortality, and it is necessary to find more biomarkers for revealing the pathogenic mechanism (Guo et al., 2022). In the present study, 39 genes that both correlated with STAD initiation and clinical traits of patients were initially extracted. The overlapping genes were mainly enriched in prion diseases and the biosynthesis of unsaturated fatty acids. Prions have adhesion molecular properties that promote the development, progression, and epithelial–mesenchymal transition of tumors by affecting the ERK2 signaling pathway. Moreover, prions were highly expressed in gastric, pancreatic, colon, and breast cancers. They can enhance cancer cell metastasis and proliferation and induce drug resistance (Diarra-Mehrpour et al., 2004; Pan et al., 2006; Du et al., 2013). Regarding the unsaturated fatty acid pathway, a large number of studies have reported that abnormal lipid metabolism was related to the occurrence and development of tumors (Menendez and Lupu, 2007). Tumors generally stimulate the fatty acid synthase to promote lipid synthesis and meet the lipid requirement for tumor cell proliferation (Kim and DeBerardinis, 2016). The synthesis of unsaturated fatty acids in tumor cells leads to lipid peroxidation and mitochondrial damage, resulting in mitochondrial DNA leakage, ultimately activating the cGAS-STING innate immune pathway (Xiang et al., 2023). cGAS-STING is the main pathway responsible for recognizing cytoplasmic DNA immune responses. As a cytoplasmic DNA receptor, cGAS can be activated by DNA and/or Mn2+ to synthesize the second messenger 2′3′-cGAMP using ATP and GTP, which further activates STING and induces the production of type I interferons and other cytokines, thereby eliciting tumor immune responses (Chen and Xu, 2023). Additionally, research indicates a correlation between the unsaturated fatty acid synthesis pathway and ferroptosis. Long-chain fatty acid-CoA ligase 4 (ACSL4) and lysophosphatidylcholine acyltransferase 3 (LPCAT3) facilitate the binding of polyunsaturated fatty acids (PUFAs) to phospholipids, forming phospholipids containing PUFAs (PUFA–PLs), which are susceptible to oxidation mediated by lipoxygenases (ALOX), resulting in iron-dependent regulatory cell death (Chen et al., 2021). Studies have shown that in gastric cancer cells, the expressions of long-chain fatty acid protein 5 (ELOVL5) and fatty acid desaturase 1 (FADS1) discriminate the cellular susceptibility to ferroptosis (Lee et al., 2020).

Furthermore, three genes, namely, *PIP5K1P1*, *PTTG3P*, and *SNORD15B*, were determined via the LASSO analysis. Moreover, three genes’ expression affected the STAD patients’ prognosis significantly. *PIP5K1P1*, phosphatidylinositol-4-phosphate 5-kinase type 1 pseudogene 1, resembles the *PIP5K1A* functional gene that plays a role in the upstream of the P13K/AKT signaling pathway and is involved in cell differentiation, cell migration, and so on. Pseudogenes are fragments that are very similar to known sequences of coding genes called parent or true genes. Pseudogenes lack the function of encoding a protein or peptide due to premature stop codons, deletions, insertions, and mutations. Therefore, it was once considered to be an insignificant gene fragment in biomolecular processes (Pei et al., 2012). However, there are currently no studies on the role of this gene in tumors, and its mechanism of action in tumors needs to be further explored. *PTTG3P*, pituitary tumor transforming 3, pseudogene, is a member of the PTTG family, and its parents are PTTG1 and PTTG2. It is involved in the progression of many cancers. PTTG3P promoted cell proliferation and glycolysis in colorectal cancer (Wang et al., 2021) and also promoted metastasis by sponge-absorbing microRNA-155-5P (Liu et al., 2020). In breast cancer, there is a positive relationship between PTTG3P and PTTG1 expression, with high expression indicating a poor prognosis (Lou et al., 2019). In this work, we found that its low expression favored prognosis in STAD. This result is identical to the previously reported research. Weng et al. (2017) indicated that the expression of the pseudogene PTTG3P was higher in stomach tumors than in normal tissues and that high expression of PTTG3P facilitated proliferation, migration, and invasion of gastric tumor cells and was associated with poor prognosis (Weng et al., 2017). This is because PTTG3P can upregulate the *YAP1* gene, which can promote cell growth (Johnson and Halder, 2014; Shimomura et al., 2014) and inhibit apoptosis (Zhao et al., 2009). SNORD15B is a C/D box snoRNA encoded in the ribosomal protein S3 gene (Tycowski et al., 1993). Some snoRNAs are the impact of the tumor of proliferation, clonality, migration, and invasion in some previous studies (Zhang et al., 2020). In this work, its lower expression showed a better prognosis for patients with STAD.

In addition, we also found that pathways related to cancer cell proliferation (oxidative phosphorylation and RNA polymerase) were enriched in the SNORD15B high-expression group. This may be because it enhances the proliferation and colony formation of stomach tumor cells via those pathways (Shen et al., 2022).

Based on three genes, we first built the risk score model. The low-risk score was helpful for STAD patient prognosis and demonstrated a good predictive capability for patients with STAD. Consistent with this, the KM plot indicated that a low-risk score was associated with better prognosis for STAD patients when grouped by gender, age, and stage. Moreover, the three-gene risk score was proved to be an independent prognostic risk factor of STAD via Cox regression analyses. In addition, we found an interaction association between the risk score and N and clinical stages, with the N stage mediating the effect of the risk score on the prognosis of the patients with STAD. We speculated that lymph node metastasis may be related to the elevation of the risk score. Finally, a nomogram based on the three-gene risk score and other clinical features (G, M, N, T, stage, sex, and age) was constructed for predicting the prognosis of patients with STAD.

Compared with published STAD prognosis prediction models, including the three-gene model established by Wu et al. (2018), the five-gene model by Song et al. (2019), the eight-gene model by Wei et al. (2020), the four-gene model by Guo et al. (2022), and the four-gene model by Fu et al. (2022), we found no overlap in key genes between our model and theirs. The differences in the genes identified may be due to variations in gene screening processes and the types of genes emphasized. For instance, Fu et al. (2022) focused on mutation-related prognostic genes, while Guo et al. (2022) focused on invasion-related genes. We compared the C-index of the models [Guo et al. (0.62) vs. Wu et al. (0.57) vs. Song et al. (0.58) vs. Wei et al. (0.54) vs. Fu et al. (0.59) vs. our study (0.61)] and the results of the time-dependent ROC curve in predicting the 1-year survival rate of patients. Our AUC ranked second, just behind Fu et al.’s study [AUC of 1 year: Guo et al. (0.62) vs. Wu et al. (0.58) vs. Song et al. (0.60) vs. Wei et al. (0.51) vs. Fu et al. (0.64) vs. our study (0.61)]. In predicting the 3-year survival rate of patients, our AUC was the highest [3-year: Guo et al. (0.66) vs. Wu et al. (0.58) vs. Song et al. (0.66) vs. Wei et al. (0.60) vs. Fu et al. (0.64) vs. our study (0.71)]. In predicting the 5-year survival rate, our AUC was very close to Guo’s, both exceeding 0.8 [5-year: Guo et al. (0.82) vs. Wu et al. (0.62) vs. Song et al. (0.66) vs. Wei et al. (0.60) vs. Fu et al. (0.64) vs. our study (0.81)]. Those results indicated that our model showed good performance, and with fewer key genes, it is relatively easier to detect.

The three-gene model might play a vital role in the prognosis of patients with STAD by regulating important pathways. It can assess the prognosis of gastric cancer patients and provide a reference for the development of subsequent treatment plans. Additionally, we identified *PIP5K1P1* as a pseudogene. Pseudogenes were previously considered useless genes, resulting in limited research on them. This study indicates that pseudogenes also play an important role in the occurrence and development of diseases and should be given more attention. However, this study has some limitations. First, there was a limited sample size. Second, the TCGA database is not comprehensive because that database only contains patients from America. Third, we did not consider incorporating potential confounding factors, including lifestyle factors (for example, smoking and alcohol consumption) and comorbidities, due to a lack of that information from the TCGA database. In addition, we are not conditioned to collect this information from patients. Fourth, there was no experiment to study the function of three genes in STAD-related signaling pathways because of the lack of clinical patients and realistic conditions. Furthermore, the specific regulatory mechanisms of three genes for STAD need to be further explored experimentally. Therefore, our future research will focus on validating the conclusions of this study, both in terms of clinical application and molecular mechanisms.

## 5 Conclusion

In this study, the constructed three-gene signature (*PIP5K1P1*, *PTTG3P*, and *SNORD15B*) has an independent predictive probability for prognosis. It can provide a model to predict the prognosis for patients with STAD. Specific related mechanisms need to be further explored.

## Data Availability

The raw data supporting the conclusions of this article will be made available by the authors, without undue reservation.
