# Supplementary figures and images for: Construction of a prognostic model via WGCNA combined with the LASSO algorithm for stomach adenocarcinoma patients
Source: Front Genet. 2024 Aug 7;15:1418818. doi: 10.3389/fgene.2024.1418818 (PMC11335515; doi:10.3389/fgene.2024.1418818)

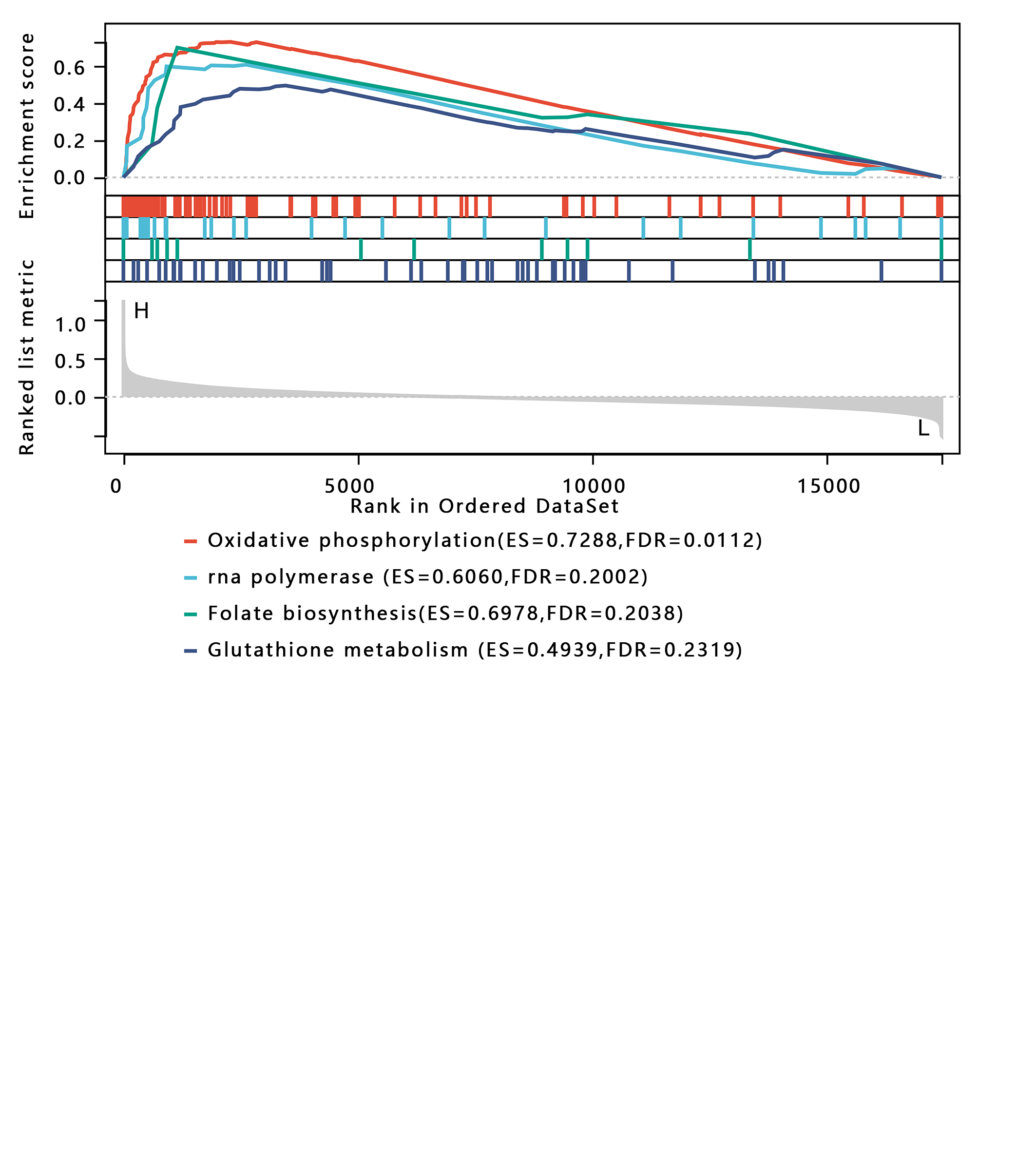

Supplement: Supplementary file 1 [file Image6.TIF]

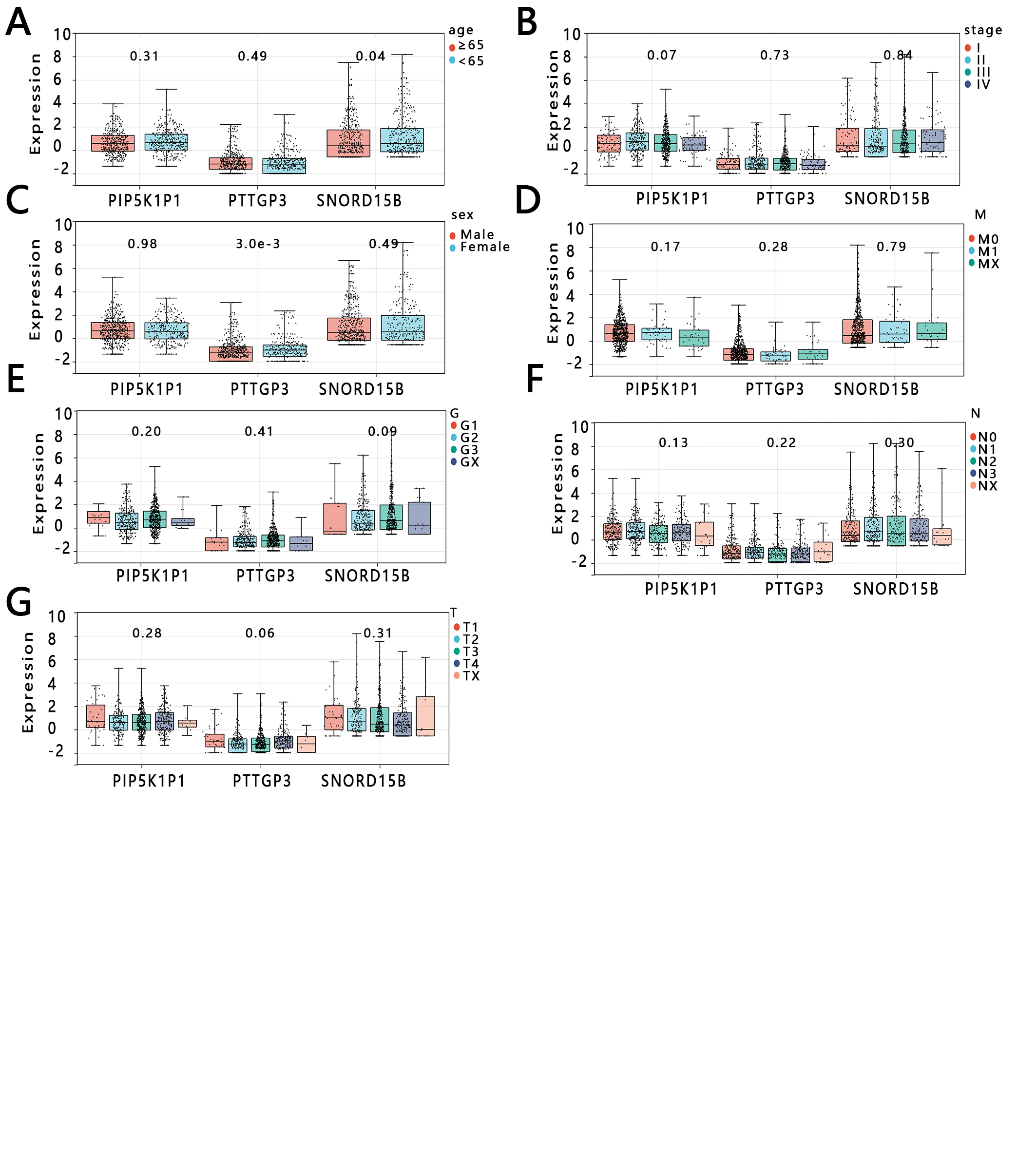

Supplement: Supplementary file 3 [file Image3.TIF]

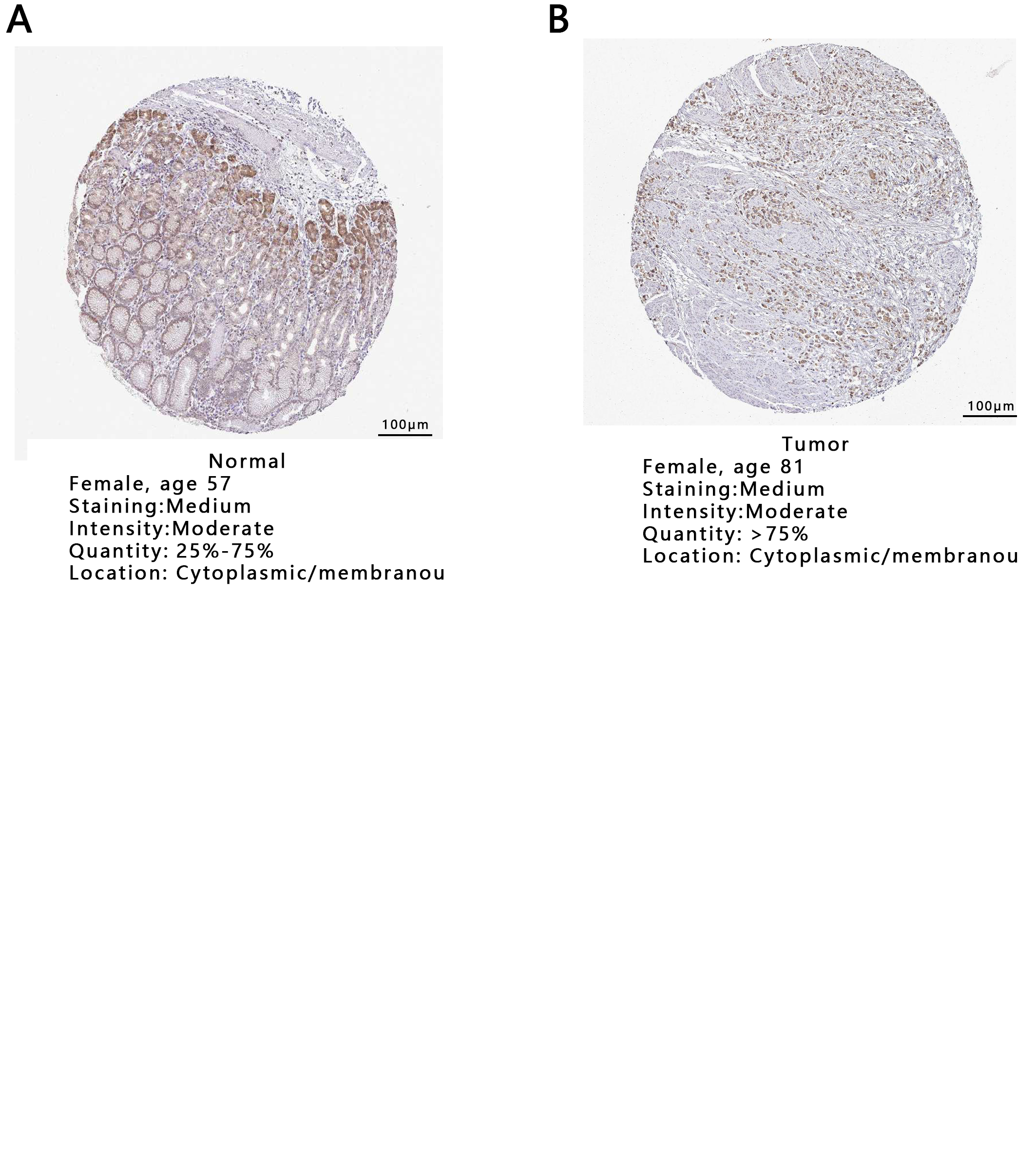

Supplement: Supplementary file 4 [file Image4.TIF]

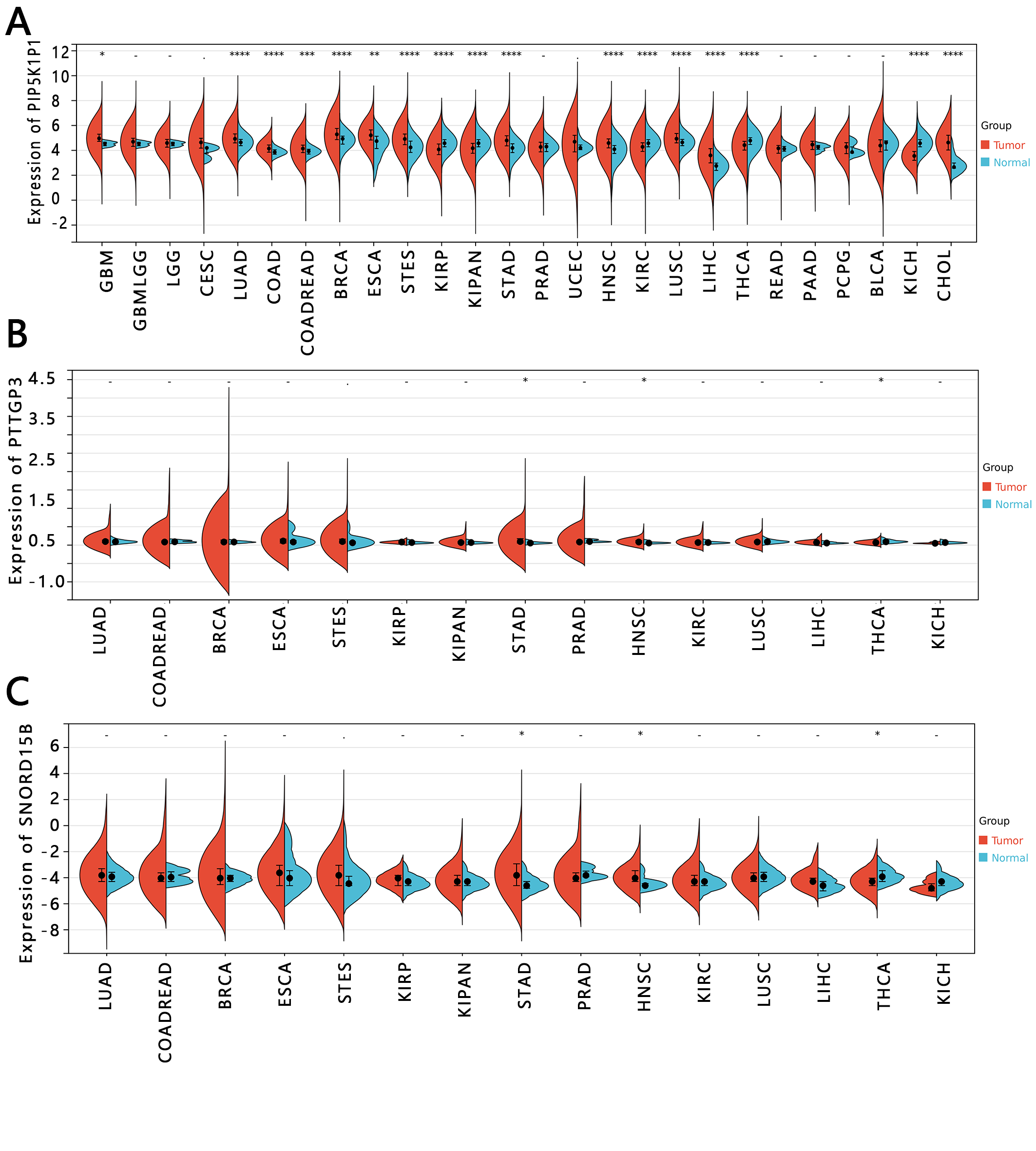

Supplement: Supplementary file 5 [file Image2.TIF]

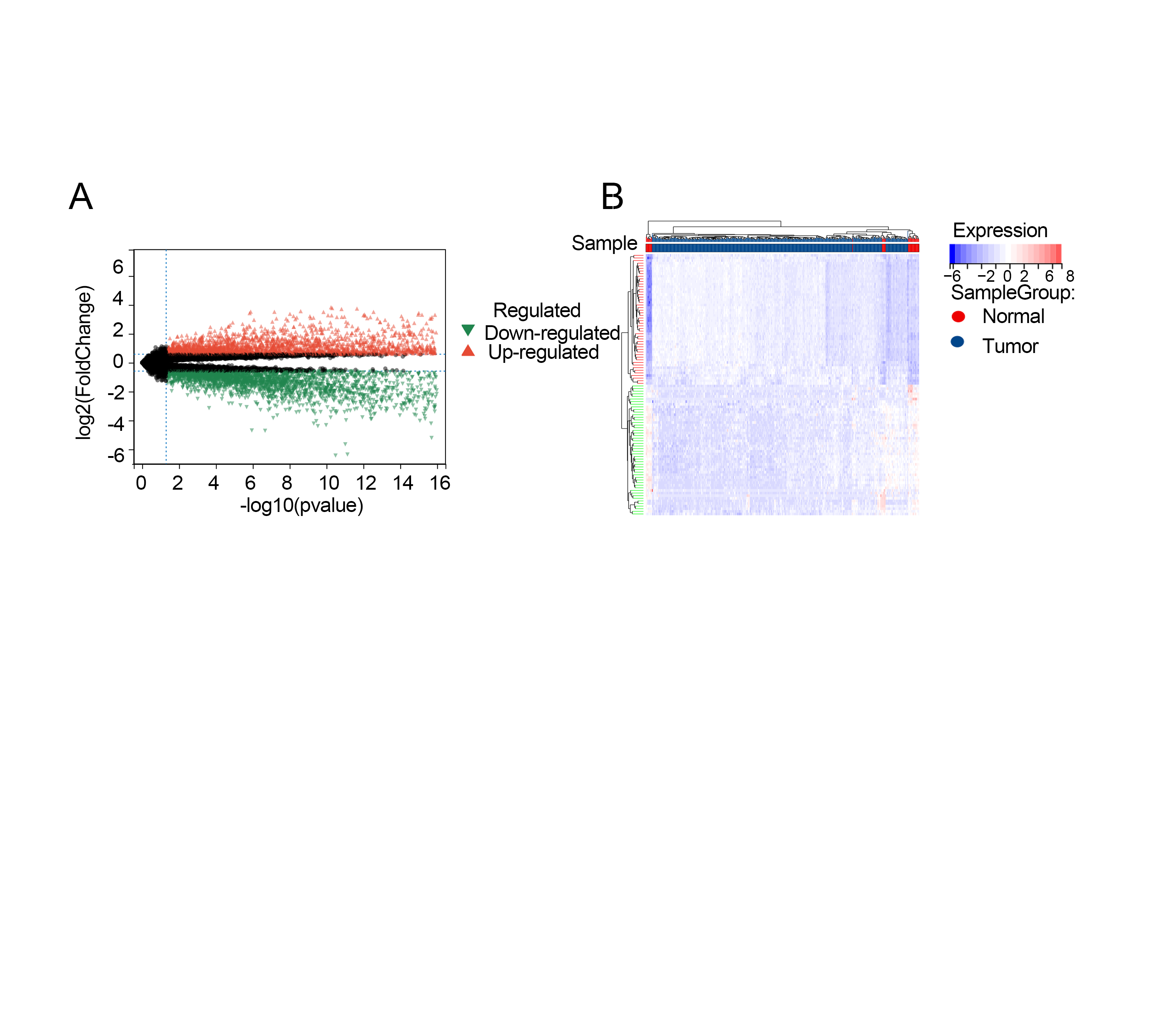

Supplement: Supplementary file 6 [file Image1.TIF]

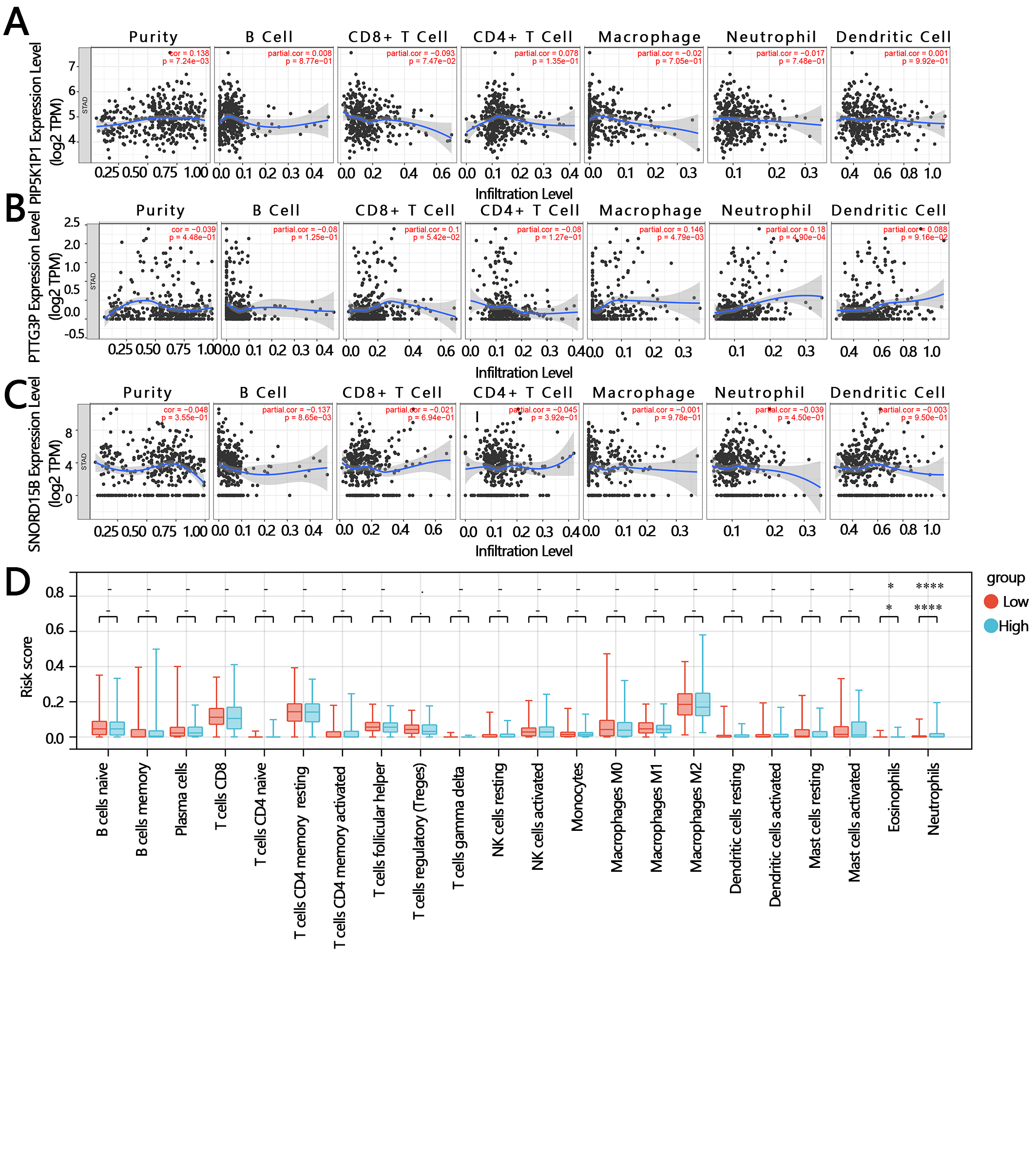

Supplement: Supplementary file 7 [file Image7.TIF]

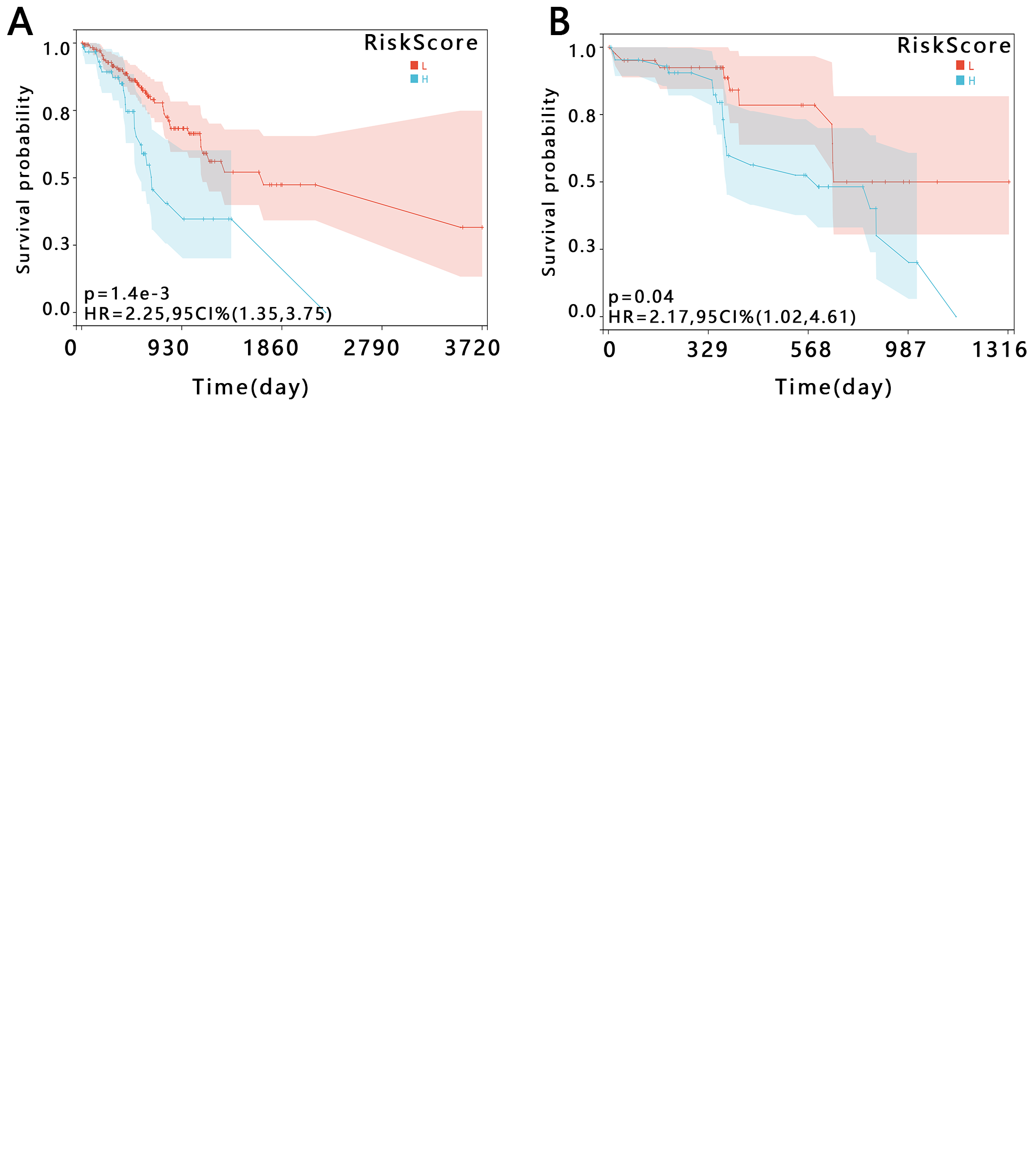

Supplement: Supplementary file 10 [file Image5.TIF]
