# Supplementary material for: Construction of a prognostic model via WGCNA combined with the LASSO algorithm for stomach adenocarcinoma patients
Source: Front Genet. 2024 Aug 7;15:1418818. doi: 10.3389/fgene.2024.1418818 (PMC11335515; doi:10.3389/fgene.2024.1418818)
Supplement: Supplementary file 2 [file Table1.docx]

**Table S1** The survival analysis of PIP5K1P1 in different cancers

| Type of cancer | KM analysis | | | Cox analysis | | |
| --- | --- | --- | --- | --- | --- | --- |
|  | HR | 95%CI | P | HR | 95%CI | P |
| KIPAN | 2.160 | 1.019-4.575 | 0.067 | 2.200 | 0.927-5.241 | 0.074 |
| BLCA | 0.717 | 0.528-0.973 | 0.026 | 0.716 | 0.532-0.963 | 0.027 |
| BRCA | 1.780 | 1.203-2.620 | 0.014 | 1.800 | 1.118-2.894 | 0.016 |
| CESC | 1.880 | 0.973-3.614 | 0.133 | 1.880 | 0.813-4.343 | 0.140 |
| CHOL | 0.000 | 0.000-0.000 | 0.187 | <0.001 | 0.000-inf | 0.998 |
| KICH | 0.287 | 0.083-0.996 | 0.093 | 0.287 | 0.061-1.353 | 0.115 |
| KIRC | 2.150 | 1.395-3.319 | <0.001 | 2.150 | 1.530-3.032 | <0.001 |
| COADREAD | 0.745 | 0.464-1.195 | 0.226 | 0.741 | 0.456-1.206 | 0.228 |
| UCEC | 2.020 | 1.199-3.419 | 0.019 | 2.030 | 1.112-3.695 | 0.021 |
| ESCA | 3.300 | 1.456-7.490 | 0.069 | 3.490 | 0.838-14.507 | 0.086 |
| GBM | 1.440 | 0.984-2.105 | 0.040 | 1.460 | 1.013-2.111 | 0.042 |
| HNSC | 2.470 | 1.375-4.418 | 0.038 | 2.480 | 1.021-6.038 | 0.045 |
| LIHC | 1.310 | 0.922-1.863 | 0.143 | 1.310 | 0.910-1.899 | 0.144 |
| LGG | 1.800 | 0.781-4.163 | 0.069 | 1.820 | 0.947-3.480 | 0.073 |
| LUAD | 0.831 | 0.622-1.112 | 0.207 | 0.831 | 0.624-1.108 | 0.208 |
| LUSC | 1.290 | 0.867-1.915 | 0.167 | 1.290 | 0.898-1.857 | 0.168 |
| PAAD | 0.739 | 0.492-1.110 | 0.148 | 0.738 | 0.489-1.115 | 0.149 |
| KIRP | 2.150 | 0.596-7.746 | 0.098 | 2.170 | 0.848-5.549 | 0.106 |
| THCA | 0.159 | 0.054-0.465 | 0.041 | 0.159 | 0.021-1.201 | 0.075 |
| PCPG | 5.690 | 0.918-35.267 | 0.022 | 5.920 | 1.068-32.815 | 0.042 |
| PRAD | 0.420 | 0.094-1.881 | 0.160 | 0.408 | 0.113-1.478 | 0.172 |
| READ | 5.440 | 0.771-38.356 | 0.003 | 6.190 | 1.602-23.913 | 0.008 |
| UCEC | 0.525 | 0.210-1.315 | 0.088 | 0.518 | 0.240-1.116 | 0.093 |
| GBMLGG | 0.205 | 0.071-0.591 | 0.084 | 0.202 | 0.027-1.505 | 0.118 |
| STES | 0.181 | 0.047-0.703 | 0.004 | 0.154 | 0.038-0.632 | 0.009 |

Abbreviation: KM: Kaplan-Mare; HR: Hazard ratio; CI: Confidence Interval; GBM Glioblastoma multiforme; GBMLGG glioblastoma and low-grade glioma; LGG Brain Lower Grade Glioma; CESC Cervical squamous cell carcinoma and endocervical adenocarcinoma; LUAD Lung adenocarcinoma; COAD Colon adenocarcinoma; COADREAD colorectal cancer; BRCA Breast invasive carcinoma; ESCA Esophageal carcinoma; STES Stomach and Esophageal carcinoma; KIRP Kidney renal papillary cell carcinoma; KIPAN Pan-kidney cohort; PRAD Prostate adenocarcinoma; UCEC Uterine Corpus Endometrial Carcinoma; HNSC Head and Neck squamous cell carcinoma; KIRC Kidney renal clear cell carcinoma; LUSC Lung squamous cell carcinoma; LIHC Liver hepatocellular carcinoma; THCA Thyroid carcinoma; READ Rectum adenocarcinoma; PAAD Pancreatic adenocarcinoma; PCPG Pheochromocytoma and Paraganglioma; BLCA Bladder Urothelial Carcinoma; KICH Kidney Chromophobe; CHOL Cholangio carcinoma.
