# Supplementary material for: Construction of a prognostic model via WGCNA combined with the LASSO algorithm for stomach adenocarcinoma patients
Source: Front Genet. 2024 Aug 7;15:1418818. doi: 10.3389/fgene.2024.1418818 (PMC11335515; doi:10.3389/fgene.2024.1418818)
Supplement: Supplementary file 8 [file Table2.docx]

**Table S2** The survival analysis of PTTG3P in different cancers

| Type of cancer | KM analysis | | | Cox analysis | | |
| --- | --- | --- | --- | --- | --- | --- |
|  | HR | 95%CI | P | HR | 95%CI | P |
| KIPAN | 6.700 | 2.592-17.294 | <0.001 | 7.101 | 3.284-15.360 | <0.001 |
| BRCA | 1.570 | 1.141-2.170 | 0.005 | 1.580 | 1.143-2.172 | 0.006 |
| KICH | 9.850 | 2.038-47.555 | <0.001 | 9.890 | 2.554-38.276 | 0.001 |
| KIRC | 2.350 | 1.617-3.406 | <0.001 | 2.361 | 1.732-3.210 | <0.001 |
| COADREAD | 0.597 | 0.370-0.965 | 0.027 | 0.582 | 0.356-0.944 | 0.029 |
| ESCA | 1.670 | 1.067-2.606 | 0.024 | 1.733 | 1.071-2.792 | 0.025 |
| NHSC | 1.370 | 1.000-1.878 | 0.072 | 1.370 | 0.970-1.939 | 0.074 |
| LIHC | 2.450 | 1.613-3.731 | <0.001 | 2.460 | 1.733-3.502 | <0.001 |
| LUAD | 1.580 | 1.142-2.178 | 0.002 | 1.580 | 1.174-2.128 | 0.003 |
| LUSC | 0.600 | 0.401-0.900 | 0.003 | 0.595 | 0.422-0.838 | 0.003 |
| KIRP | 6.170 | 2.312-16.441 | <0.001 | 6.220 | 3.403-11.368 | <0.001 |
| THCA | 0.457 | 0.171-1.216 | 0.135 | 0.455 | 0.158-1.312 | 0.145 |
| PRAD | 6.340 | 0.498-80.757 | 0.002 | 7.020 | 1.672-29.471 | 0.008 |
| STES | 1.310 | 0.955-1.786 | 0.088 | 1.310 | 0.960-1.791 | 0.089 |

Abbreviation: KM: Kaplan-Mare; HR: Hazard ratio; CI: Confidence Interval; KIPAN Pan-kidney cohort; BRCA Breast invasive carcinoma; KICH Kidney Chromophobe; KIRC Kidney renal clear cell carcinoma; COADREAD colorectal cancer; ESCA Esophageal carcinoma; HNSC Head and Neck squamous cell carcinoma; LIHC Liver hepatocellular carcinoma; LUAD Lung adenocarcinoma; LUSC Lung squamous cell carcinoma; KIRP Kidney renal papillary cell carcinoma; THCA Thyroid carcinoma; PRAD Prostate adenocarcinoma; STES Stomach and Esophageal carcinoma.
