# Supplementary material for: Construction of a prognostic model via WGCNA combined with the LASSO algorithm for stomach adenocarcinoma patients
Source: Front Genet. 2024 Aug 7;15:1418818. doi: 10.3389/fgene.2024.1418818 (PMC11335515; doi:10.3389/fgene.2024.1418818)
Supplement: Supplementary file 9 [file Table3.docx]

**Table S3** The survival analysis of SNORD15B in different cancers

| Type of cancer | KM analysis | | | Cox analysis | | |
| --- | --- | --- | --- | --- | --- | --- |
|  | HR | 95%CI | P | HR | 95%CI | P |
| KIPAN | 1.690 | 0.381-7.491 | 0.382 | 1.701 | 0.508-5.682 | 0.389 |
| BRCA | 0.857 | 0.576-1.274 | 0.466 | 0.857 | 0.565-1.299 | 0.467 |
| KICH | 4.370 | 0.497-38.454 | 0.019 | 4.382 | 1.132-16.976 | 0.032 |
| KIRC | 1.890 | 1.355-2.631 | <0.001 | 1.892 | 1.401-2.555 | <0.001 |
| COADREAD | 0.472 | 0.225-0.987 | 0.134 | 0.471 | 0.171-1.292 | 0.143 |
| ESCA | 1.520 | 0.856-2.686 | 0.105 | 1.520 | 0.912-2.537 | 0.108 |
| NHSC | 1.120 | 0.827-1.528 | 0.441 | 1.121 | 0.834-1.517 | 0.441 |
| LIHC | 0.372 | 0.208-0.666 | 0.024 | 0.372 | 0.152-0.909 | 0.030 |
| LUAD | 1.500 | 0.901-2.511 | 0.063 | 1.510 | 0.975-2.353 | 0.065 |
| LUSC | 0.730 | 0.544-0.981 | 0.051 | 0.729 | 0.530-1.003 | 0.052 |
| KIRP | 1.270 | 0.663-2.440 | 0.446 | 1.280 | 0.681-2.391 | 0.447 |
| THCA | 2.890 | 0.578-14.472 | 0.054 | 2.910 | 0.934-9.038 | 0.065 |
| PRAD | 4.530 | 1.308-15.682 | 0.034 | 4.633 | 0.975-21.935 | 0.054 |
| STES | 0.182 | 0.017-1.991 | 0.004 | 0.142 | 0.031-0.649 | 0.012 |

Abbreviation: KM: Kaplan-Mare; HR: Hazard ratio; CI: Confidence Interval; KIPAN Pan-kidney cohort; BRCA Breast invasive carcinoma; KICH Kidney Chromophobe; KIRC Kidney renal clear cell carcinoma; COADREAD colorectal cancer; ESCA Esophageal carcinoma; HNSC Head and Neck squamous cell carcinoma; LIHC Liver hepatocellular carcinoma; LUAD Lung adenocarcinoma; LUSC Lung squamous cell carcinoma; KIRP Kidney renal papillary cell carcinoma; THCA Thyroid carcinoma; PRAD Prostate adenocarcinoma; STES Stomach and Esophageal carcinoma.
